# Eleven Crucial Pesticides Appear to Regulate Key Genes That Link MPTP Mechanism to Cause Parkinson’s Disease through the Selective Degeneration of Dopamine Neurons

**DOI:** 10.3390/brainsci13071003

**Published:** 2023-06-28

**Authors:** Athira Anirudhan, George Chandy Mattethra, Khalid J. Alzahrani, Hamsa Jameel Banjer, Fuad M. Alzahrani, Ibrahim F. Halawani, Shankargouda Patil, Ashutosh Sharma, Prabu Paramasivam, Shiek S. S. J. Ahmed

**Affiliations:** 1 Central Research Laboratory, Believers Church Medical College Hospital, Kuttapuzha, Thiruvalla 689103, Kerala, India; 2 Department of Clinical Laboratories Sciences, College of Applied Medical Sciences, Taif University, P.O. Box 11099, Taif 21944, Saudi Arabia; 3 College of Dental Medicine, Roseman University of Health Sciences, South Jordan, UT 84095, USA; 4 Regional Department of Bioengineering, NatProLab-Plant Innovation Lab, Tecnologico de Monterrey, Queretaro 76130, Mexico; 5 School of Medicine, Department of Neurology, University of New Mexico Health Sciences Center, University of New Mexico, Albuquerque, NM 87131, USA; 6 Drug Discovery & Omics Lab, Chettinad Hospital and Research Institute, Chettinad Academy of Research and Education, Kelambakkam 603103, Tamil Nadu, India

**Keywords:** Parkinson’s disease, systems biology approach, pesticide toxicity, docking, blood–brain barrier (BBB), neurodegeneration

## Abstract

Pesticides kill neurons, but the mechanism leading to selective dopaminergic loss in Parkinson’s disease (PD) is unknown. Understanding the pesticide’s effect on dopaminergic neurons (DA) can help to screen and treat PD. The critical uptake of pesticides by the membrane receptors at DA is hypothesized to activate a signaling cascade and accelerate degeneration. Using MPTP as a reference, we demonstrate the mechanisms of eleven crucial pesticides through molecular docking, protein networks, regulatory pathways, and prioritization of key pesticide-regulating proteins. Participants were recruited and grouped into control and PD based on clinical characteristics as well as pesticide traces in their blood plasma. Then, qPCR was used to measure pesticide-associated gene expression in peripheral blood mononuclear cells between groups. As a result of molecular docking, all eleven pesticides and the MPTP showed high binding efficiency against 274 membrane receptor proteins of DA. Further, the protein interaction networks showed activation of multiple signaling cascades through these receptors. Subsequent analysis revealed 31 biological pathways shared by all 11pesticides and MPTP that were overrepresented by 46 crucial proteins. Among these, CTNNB1, NDUFS6, and CAV1 were prioritized to show a significant change in gene expression in pesticide-exposed PD which guides toward therapy.

## 1. Introduction

Parkinson’s disease (PD) is a complex movement disorder that affects the elderly population worldwide. PD is characterized by the loss of dopaminergic (DA) neurons in the substantia nigra (SNC) region of the human brain. Degeneration of DA showscardinal symptoms such as bradykinesia, tremor, rigidity, and postural instability. Several risk factors, including age, gender, and genetics, have been reported to cause PD. However, the age and genetic factors have a strong association with PD [1]. Additionally, it is suggested that environmental factors have the ability to trigger the degeneration of dopaminergic neurons and develop PD [2,3,4]. For instance, 1-methyl-4-phenyl-1,2,3,6-tetrahydropyridine (MPTP) is a chemical substance that has long been known for its involvement in the degeneration of dopaminergic neurons, resulting in similar clinical features toPD [5]. According to the findings of Verma A et al., 2023 andRudenok M et al., 2022 [6,7], MPTP alter the neuronal gene expression to cause Parkinson’s disease. Meanwhile, numerous epidemiological and toxicological studies in recent years have suggested that pesticide exposures such as rotenone, maneb, dieldrin, heptachlor, atrazine, and pyrethroids are linked to alpha-synuclein accumulation and dopaminergic neurodegeneration [6,7,8,9]. However, their mechanism for causing neurodegeneration is largely unknown. Understanding the pesticide-related PD pathogenesis may aid in the identification of early markers for screening, prevention, and management of PD.

Most pesticides share similar physicochemical properties with MPTP, which could be the cause of PD. Notably, pesticides and MPTP can readily cross the blood–brain barrier due to their lipophilic nature and induce oxidative stress, dysregulate dopamine transporters, alter mitochondrial function, and stimulate neuroinflammation and fibrillation to cause neuronal damage [10]. Particularly, organophosphates inhibit cholinesterase and sodium channels in the central nervous system to cause neurodegeneration [11]. Nonetheless, the molecular mechanism underlying the selective loss of dopaminergic neurons at SNC remains largely unknown. We postulate that pesticide-associated membrane receptors at dopaminergic neurons and their activation will influence degeneration. For instance, pesticides are observed to activate G protein-coupled receptors and initiate a cascade of molecular events, including the PI3K/AKT signaling pathway, to promote neurodegeneration [12,13,14]. Though various pesticides have been reported in PD, there have not been many investigations on the pesticide–receptors-mediated neurodegeneration in PD. Therefore, this study aimed to comprehend the interaction between a pesticide and a membrane receptor that signals to induce degeneration in DA neurons to cause PD. 

**Figure 1:**
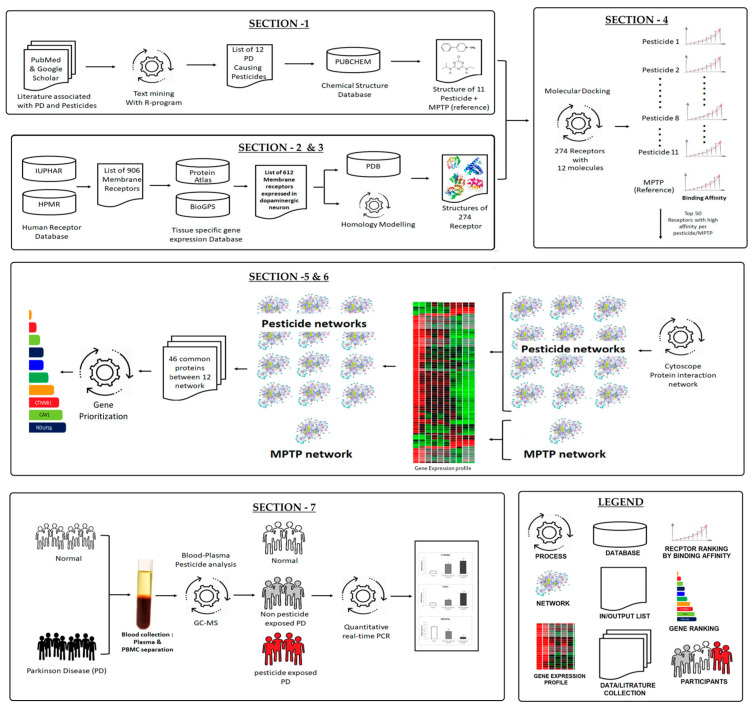
Systematic workflow demonstrating the stepwise process of our approach.

Recently, the systems biology approach of integrating the varieties of multi-omics large-scale data has provided a solution to understand the behavior of complex diseases. Notably, the systems biological approach of the protein network has shown promise in discovering novel pathological events across diseases. Moreover, it provides a novel way to understand molecular behavior in diseases [15]. This approach contributes to the identifying of novel diagnostic markers, drug targets, and therapeutic drugs [16]. Thus, using a systems biology approach, it is possible to investigate the dopaminergic neuronal membrane receptors for the acceptance of crucial pesticides and the activation of molecular events that could shed light on pesticide mechanisms in PD. Herein, our systems biological strategies (Figure 1) include integration computational concepts such as molecular docking, pesticide–receptor protein network construction, selecting pesticide-associated crucial protein-encoding genes from the network, and validating through gene expression in peripheral mononuclear cells (PBMC) of Parkinson’s disease patients and compared with healthy controls. Overall, this study demonstrates the behavior of 11 critical pesticides that cause selective degeneration of DA neurons and that behave similarly to MPTP to cause Parkinson’s disease.

## 2. Materials and Methods

A systematic workflow (Figure 1) projects an overview of our approach. The workflow was divided into seven sections: Section 1: The list of PD-associated pesticides was gathered from the literature using a text-mining approach with the R-programming language, and its chemical structures (n = 11 pesticides + 1 MPTP) were downloaded from the PUBCHEM compound database. Section 2: Using the receptor databases, a list of human membrane receptors was compiled, and their expression in dopaminergic neurons (DA) was investigated. Section 3: Based on availability, the protein structures of membrane receptors expressed in DA (list from Section 2) were retrieved from the Protein Data Bank and the remaining was generated with a homology-modeled method. Section 4: Each pesticide as well as the MPTP was docked against the collected receptor protein structure. Based on the binding energy, only the top50 receptors for each pesticide were chosen to build a pesticide–receptor protein network. Section 5: Overall, 12 networks were generated. To ascertain that each protein network was regulated in DA neurons, the genes that are expressed in dopaminergic neurons were retained in the network. Section 6: Then, the common proteins between the 12 networks (11 pesticides and 1MPTP reference) were identified and prioritized based on their functional importance. Section 7: Finally, the top3 prioritized protein encoding genes were validated in the peripheral blood mononuclear cell (PBMC) of the participants who were classified as: (1) healthy controls; (2) PD patients; and (3) PD with pesticide exposure, based on the neurological evaluation and gas chromatography-mass spectroscopy (GC-MS).

### 2.1. Text Mining and Pesticide Structure Collection 

A systematic literature search was performed to collect relevant PubMed and Google Scholar abstracts between the years 1980 and 2021. The search strategy identified the eligible abstracts using a variety of keywords related to “Parkinson’s disease” and “pesticide”. Additionally, the reference lists of the retrieved abstracts were manually searched by two authors to collect more relevant studies that were missed during the electronic search. The following inclusion criteria were used in selecting literature: (a) an abstract presenting both pesticides and PD; (b) studies with a case-control or cohort design; and (c) publications in English. Abstracts were excluded if they were: (a) review articles, letters to editors, or editorials; (b) not offering essential data; (c) duplicates of previously published articles. The names of the pesticides reported in the above PD-related abstracts were listed through text-mining with the R-program. Then, three-dimensional chemical structures of the listed pesticides were downloaded from the database (https://pubchem.ncbi.nlm.nih.gov/ accessed on 2 June 2021) in structure-data file (SDF) format. Additionally, the SDF format of MPTP molecule was obtained and used as a reference to compare the result obtained for each pesticide at every step of our analysis.

### 2.2. Receptors of Dopaminergic Neurons and Structure Modeling

Simultaneously, the list of membrane receptors was collected from IUPHAR (https://iuphar.org/, accessed on 18 June 2021) and HPMR (http://www.receptome.org/, accessed on 18 June 2021) databases. Then, the gathered membrane receptors from the databases were refined for their expression in the dopaminergic neuron at substantia nigra region using the BioGPS (http://biogps.org/, accessed July 2021) and Human Protein Atlas (www.proteinatlas.org, accessed on July 2021) databases. Finally, the refined list was used as input to search for the protein structure from the Protein Data Bank (PDB) (https://www.rcsb.org/, accessed on September 2021); On account of the unavailability of the protein structure, a homology modeling approach was adopted to generate protein structures using SWISS-MODEL (https://swissmodel.expasy.org/ accessed on 2 June 2021) [17]. All generated structures were verified for quality using PROCHECK (https://www.ebi.ac.uk accessed on 2 June 2021) server [18]. 

### 2.3. Molecular Docking

Molecular docking is an extensively used computer simulation protocol to visualize the conformation of a receptor–ligand complex [19,20]. Each collected DA membrane receptor protein structure was docked with the pesticide (n = 11) and the MPTP molecule. For this, all collected receptor proteins were energy minimized; the grid was then constructed around the receptor; and docking was performed with each optimized pesticide and MPTP molecule. Molecular docking was carried out with iGEMDOCK (http://gemdock.life.nctu.edu.tw/dock/igemdock.php accessed on 2 June 2021) by adopting the Generic Evolutionary Method for Molecular Docking scoring function. Docking analysis provides the lowest energy profile, indicating the high likelihood of binding interactions between the receptor protein and pesticide. Among the receptor proteins, the top fifty receptors for each pesticide were selected to construct the pesticide–receptor protein network.

### 2.4. Receptor Network Analysis Differential Genes in the Network

The selected top 50receptors for each pesticide were subjected to protein–protein interactions using Cytoscape software. A similar method was used to build the protein network for the MPTP molecule. Thus, this procedure generated 12 protein networks for further assessment. In order to maintain the relevance of the network with dopaminergic neurons, only the genes expressed in dopaminergic neurons were retained within the network. The gene expressed in dopaminergic neurons were collected from the microarray gene expression dataset (GSE20141) (http://www.ncbi.nlm.nih.gov/geo accessed on 2 June 2021). The GSE20141 contains the gene expression profile of 10PD and 8control of laser-dissected substantia nigra pars compacta neurons from a postmortem brain. The differentially expressed genes (DEGs) with an adjusted *p*-value ≤ 0.05 in PD were identified using the R-program limma package. Then, the DEGs were mapped to the network; each pesticide network contains the top 50neuronal receptors activated by pesticide with the DA neuron proteins along with the subset of proteins that are differentially expressed in the PD condition. Upon generation of the network, the identified common proteins among the 12 networks (11 pesticides and 1MPTP) were subjected to enrichment analysis. The pathway analysis was performed using the KEGG database to identify the common regulating pathways of multiple pesticides and MPTP [21]. Additionally, the common proteins were prioritized based on their molecular function using the ToppGene tool [22]. In ToppGene, the differentially expressed PD genes in GSE20141 were used as a training set by leaving the identified common genes/proteins that were input as a test set for prioritization. The encoding genes of the selected top three ranked proteins were assessed in the participant with PD and compared with healthy controls.

### 2.5. Participant Recruitment

All participants for this study were recruited at Chettinad Academy of Research and Education, Tamil Nadu, India, with the approval of the Institutional Research Ethics Boards Committee. A total of 69 participants were selected based on the inclusion and exclusion criteria. Inclusion criteria: (1) Participants of South Indian origin, (2) aged 40 to 65 years, (3) body mass index ranged between 18 and 35 kg/m^2^. For the participants with PD, the inclusion criteria were based on neurological examination by the movement disorder specialist to look for the presence of cardinal symptoms and evaluate them by the Unified Parkinson’s Disease Rating Scale (UPDRS) and Hoehn and Yahr (H&Y) scale. Alternatively, the exclusion criteria included: (1) severe systemic diseases, (2) intake of vitamins and minerals; (3) infections or surgery within six months; and (4) other neuropsychiatric diseases. All participants gave written informed consent before sample collection. Participants in the healthy group were examined by the movement disorder specialist to confirm their health status as being free of neurological or neuropsychiatric diseases. 

### 2.6. Pesticide Analysis for Samples Classification

Immediately after signing the informed consent and neurological examination, 5 mL of blood was collected from each participant. Of the 5 mL of blood, 2 mL was used to obtain blood plasma for gas chromatography and mass spectroscopy (GC-MS) analysis to determine the presence of pesticides. For GC-MS, exactly 1 mL of plasma was taken and mixed with 3 mL of 2% acidified ethyl acetate and 0.4 g of MgSO_4_. The mixture was mixed well and centrifuged at 6000 rpm for 10 min. The resultant supernatant was collected and evaporated with a Turbovap nitrogen flow evaporator from Calliper Life Sciences (Mountain View, CA, USA). The evaporated samples were resuspended with 1 mL of ethyl acetate and 50 mg of primary secondary amines (PSA) (Agilent Technologies, Santa Clara, CA, USA) for sample cleanup. The reaction mixture was mixed well and centrifuged at 8000 rpm for 10 min. After centrifugation, the supernatant was transferred and evaporated [23]. The evaporated sample was reconstituted with 10 µL of ethyl acetate, and 2 µL was injected into a GC-MS (triple-quadrupole Quantum XLS mass analyzer, Thermo Scientific, Gainesville, FL, USA) for analysis. Before sample analysis, the system was prepared, and pesticide standards (Benomyl (Catalogue #45339), Carbendazim (Catalogue #79888), Maneb (Catalogue #45554), Pesticide mixture (Catalogue #CRM46845) which includes Dichlorodiphenyltrichloroethane, Dichlorodiphenyldichloroethylene, Delidrin, Heptachlor, Heptachlor epoxide, and Lindane) (Supelco Bellefonte, PA, USA, and Sigma-Aldrich) were assessed to have standard calibration curve 0.015 to 135.00 ng/mL. The presence of pesticide trace within or above the limit of detection was classified as pesticide-exposed participant. Finally, based on the neurological examination and GC-MS evaluation of pesticides, the participants were grouped as GROUP-A: pesticide exposed PD, GROUP-B: PD with no pesticide exposure, and GROUP-C: healthy control participants.

### 2.7. Sample Processing and Gene Expression Analysis

Parallel to this, the remaining 3 mL of collected peripheral blood was used for gene expression analysis by isolating the peripheral blood mononuclear cells (PBMC) using Histopaque-1077 (Sigma-Aldrich, Burlington, MA, USA). Further, total RNA was extracted from the PBMC using the TRIzol (Invitrogen, Waltham, Massachusetts, USA) reagent to construct cDNA with the 100 ng of RNA measured using Nanodrop 2000 (Thermo Fisher Scientific, Wilmington, DE, USA). The cDNA was synthesized using the High-Capacity cDNA Reverse Transcription Kits, Thermo Fisher Scientific, Waltham, MA, USA, following the manufacturer’s protocol. All collected samples were checked for their RNA quality with A260/280, a negative control was included to make sure there was no DNA contaminating the RNA extraction process. The expression was evaluated using gene-specific primers and GAPDH as the housekeeping gene (Table 1) [24]. Then, following the TakyonTM ROX SYBR 2X Master Mix manufacturer’s protocol (Eurogentec, Seraing, Liège, Belgium), the quantitative real-time PCR (ABI-7000, Applied Biosystems, Waltham, Massachusetts, USA) was run under the following conditions: 40 cycles of activation (95 °C for 3 min), denaturation (95 °C for 10 s), and annealing (60 °C for 60 s). Finally, the relative gene expression between the groups (designated based on neurological examination and GC-MS assessment) were calculated using the 2^−∆∆Ct^ method.

### 2.8. Statistical Analysis

The suitable statistical methodologies in GraphPad Software were used to assess the demographic and clinical characteristics between the groups. The distribution of the collected numerical variables was then examined. Since all of the variables were normally distributed, parametric tests were used. For instance, the age (demographic) and gene expression data were analyzed using a one-way ANOVA with Tukey post hoc analyses for multiple comparisons between groups (GROUP-A, GROUP-B, and GROUP-C). A Student *t*-test was conducted to ascertain the statistical significance between GROUP-A and GROUP-B on the UPDRS and H&Y scale. All values are presented as the mean, standard deviation, and *p*-values < 0.05 were considered statistically significant.

## 3. Results

### 3.1. Data Retrieval

Using Preferred Reporting Items for Systematic reviews and Meta-Analyses (PRISMA) criteria, the abstracts were individually collected from the literature databases by two authors and screened for the presence of search terms related to pesticides and PD. A total of 2401 abstracts were collected and subjected to a text-mining approach using the R-program. On text-mining, eleven pesticides were reported more frequently in the titles and abstracts of the collected articles. Then, the three-dimensional structures of all eleven pesticides were retrieved from the NCBI Pubchem database using their appropriate Pubchem IDs, such as Benomyl (CID_28780), Carbendazim (CID_25429), S-methyl-N-butylthiocarbamate (CID_11137311), Dichlorodiphenyltrichloroethane (CID_13089), Dichlorodiphenyldichloroethylene (CID_3035), Delidrin (CID_969491), Heptachlor (CID_3589), Heptachlor epoxide (CID_13930), Lindane (CID_727), Maneb (CID_3032581), and Rotenone (CID_6758). Additionally, the structure of MPTP (CID_1388) was collected and used as a reference for the comparative analysis. Simultaneously, the list of human membrane receptors was retrieved from the IUPHAR and HPMR databases. Among the collected 906 human membrane receptors, 612 were expressed in dopaminergic neurons in the substantia nigra of the human brain and were determined using BioGPS (http://biogps.org/, accessed on July 2021) and the Human Protein Atlas databases (www.proteinatlas.org, accessed on 2 July 2021).

### 3.2. Structure Modeling and Molecular Docking

Three-dimensional structures of 612 receptor proteins were searched in the PDB databases. Of 612 protein structures, 148 were available in PDB, while the remaining 464 were generated based on a homology modeling approach using the SWISS-MODEL server. Among 464 receptors, only 126 had valid templates with more than >60% similarity while performing NCBI protein BLAST with PDB as the reference database. Hence, 126 receptor protein structures were generated with the templates from PDB. Further, molecular docking was performed for 12 molecules (11 pesticides and MPTP) against 274 (148 PDB structure and 126 modeled structures) receptor targets. Overall, 3288 (12 × 274) molecular docking was performed, which showed the least binding energies ranging between −18.59 to −6.9 kcal/mol. For each pesticide, the top 50 membrane receptor proteins having the least binding energies were selected and subjected to the cytoscape protein–protein interaction (PPI) network. Similarly, the MPTP receptor protein network was constructed from the top 50receptor proteins with high affinity with MPTP. The constructed network for each pesticide was termed a pesticide–receptor protein network that demonstrates the signaling events mediated by the receptors upon pesticide activation.

### 3.3. Pesticide–Receptor Protein Network

**Figure 2:**
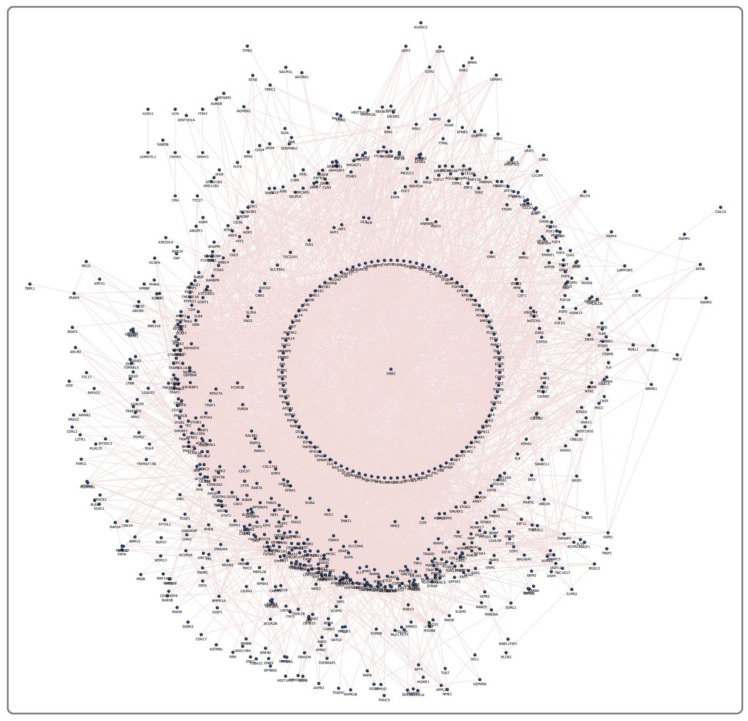
An MPTP network containing 1092 proteins with 11,145 interacting edges.

Each pesticide–receptor protein network and the MPTP receptor protein network showed the complex interaction that connects membrane receptors with multiple proteins. Each network was further refined using the GSE20141 dataset to have protein-encoding genes that were expressed by dopaminergic neurons. For instance, the MPTP receptor protein network (Figure 2) initially contained 1092 proteins (Supplementary File S1), contributing 11,145 paired interactions. Finally, on mapping with GSE20141, the MPTP network was retained with 593 proteins expressed in dopaminergic neurons. A similar network was constructed for each pesticide, demonstrating the signaling events upon pesticide activation. Furthermore, using GSE20141, the involvement of proteins in the pesticide–receptor protein network was tested for its association with PD by assessing their expression level in PD compared to the control. In the MPTP network with 593 proteins, 299 were significantly (*p* < 0.05) altered in PD. Likewise, the significant genes encoding proteins in all 11 pesticide networks were identified. Overall, 46 proteins (Table 2) were noticed to be common between the pesticides and MPTP networks.

### 3.4. Regulatory Pathway and Protein Prioritization

Enrichment of 46 common proteins showed involvement in 31 molecular pathways, including Sphingosine-1-phosphate receptor 1 (S1P1), the mTOR signaling pathway, Wnt signaling, and the ErbB receptor signaling pathways (Table 3). Additionally, these 46 common proteins were prioritized using the ToppGene Tool to select the three most contributing proteins based on their molecular functions. Therefore, CTNNB1, NDUFS6, and CAV1 were ranked in the top three, suggesting their crucial role in PD upon exposure to any of these 11 pesticides and MPTP.

### 3.5. Pesticide Detection Using GC-MS and Validation of Biomarkers

**Figure 3:**
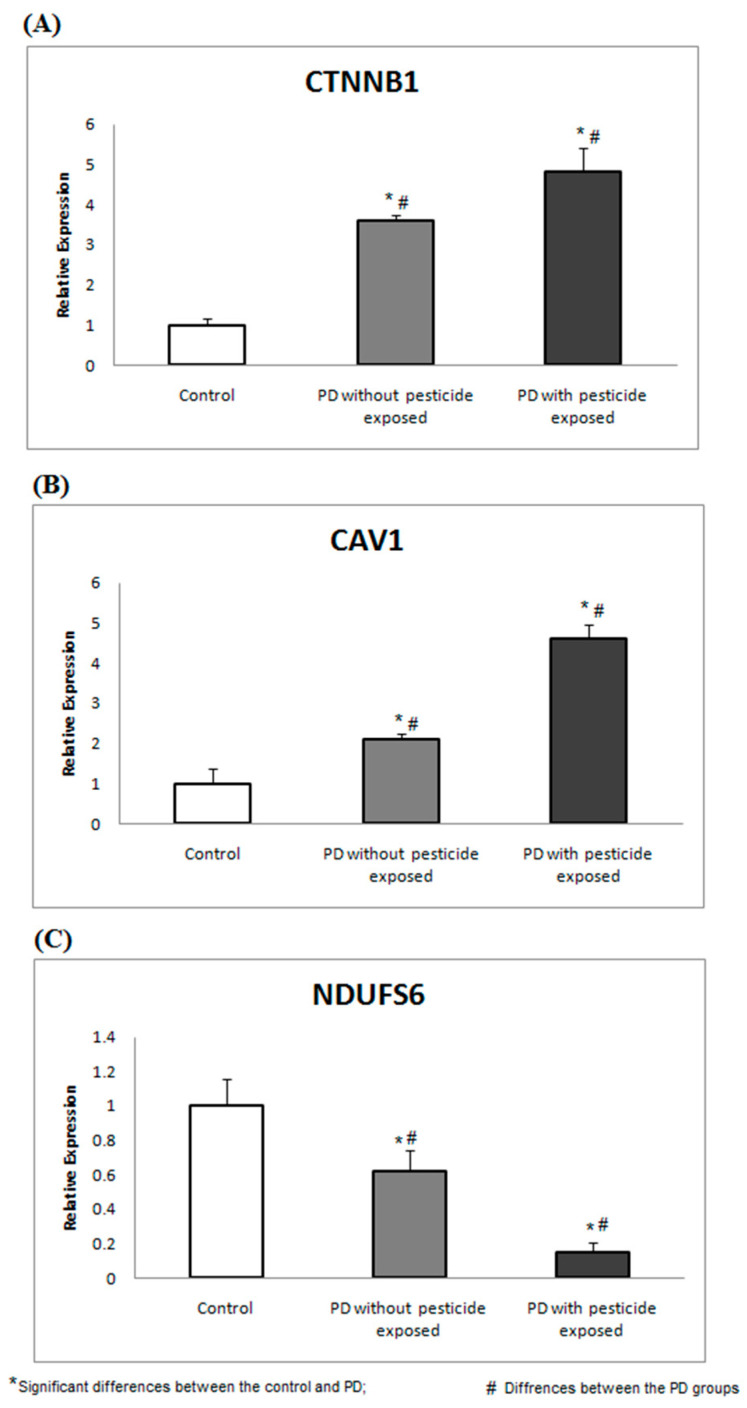
CTNNB1, CAV1, and NDUFs6 gene expressions are associated with pesticide toxicity. The expression analysis shows the altered gene expression of (**A**) CTNNB1, (**B**) CAV1, and (**C**) NDUFS6 in PD groups compared to the healthy control.

GC-MS was used to profile the presence of pesticide exposure in all 69 recruited participants. Of the recruited PD participants, 23 (GROUP-A) showed a trace of pesticide in their blood plasma, while the remainder did not show a trace of pesticide in GC-MS analysis. Particularly in GROUP-A, 61% of subjects were detected with traces of Dichlorodiphenyldichloroethylene, 26% with Dichlorodiphenyltrichloroethane, and 13% with Heptachlor. Therefore, PD participants (based on clinical evaluation) were classified as GROUP-A (n = 23). Similarly, PD participants (based on clinical evaluation) with no pesticide exposure were termed GROUP-B, containing 25 PD participants. The healthy control (confirmed clinically) with no pesticide exposure was grouped as GROUP-C (n = 21), containing 21 participants. The basic characteristics of the recruited participants were recorded (Table 4). To develop a feasible method for PD screening based on pesticide exposure, the selected topthree ranked proteins encoding genes were assessed in PBMC and compared between the groups. Using gene-specific primers (Table 1), the relative expression of CTNNB1 and CAV1 showed significant upregulation, and NDUFS6 showed downregulation (*p* < 0.05) in PD groups compared to healthy control (Figure 3).

## 4. Discussion

Pesticide exposures are a major contributing factor to PD. Several studies report that chronic pesticide/chemical exposure may cause and/or accelerate neurodegeneration. For instance, Langston JW et al. (2017) report that MPTP affects the nigrostriatal dopamine system, accelerates neuronal death [25]. Loss of dopaminergic neurons creates dopamine demand that diminishes neuronal connectivity and causes cardinal symptoms in PD [26,27,28]. Notably, most pesticides are highly lipophilic, easily cross the BBB, and accumulate in different brain regions. Subsequently, pesticides may use multiple neuronal receptors for their internal cellular transport to disrupt neuronal function [11]. Pesticides may use multiple membrane receptors expressed by the neuronal types, but the mechanism leading to the selective loss of dopamine neurons in the substantia nigra of the human brain is largely unknown. A systems biological approach was implemented in this study to demonstrate the role of pesticides in regulating genes/proteins in dopaminergic neurons that promote PD. The workflow provides a hand-in-hand comparison between the pesticides and MPTP through sequential analysis: (1) Screening of more vulnerable membrane receptors of dopaminergic neurons at the substantia nigra for each pesticide; (2) signaling events mediated by the top fifty receptors on pesticide binding; (3) key genes encoding proteins in the signaling events connecting multiple pesticides; (4) molecular pathways attributed to the signaling events; (5) key proteins involved in exposure to pesticides; (6) validation of protein-encoding genes in PBMC across the study groups. Although the regulation of each pesticide is important and enormous, we try to explain the regulatory behavior of the pesticide by comparing with the MPTP mechanism.

MPTP is a lipophilic compound that readily crosses the BBB to enter neuronal cells [27,29]. In neurons, lysosomes uptake MPTP and use the monoamino oxidase (MAO-B) enzyme to convert it into MPP+ [29]. MPP+ dysregulates neuronal function and plasticity [5]. Meredith GE. et al. 2011 report the accumulation of MPP+ in the brain region, which leads to neuropathological changes in mice [30]. MPP+ causes neuroinflammation, vacuolation of nerve cells, neuronal loss at the striatum and globus pallidus, and death of nigrostriatal dopaminergic neurons [30,31]. In this analysis, all 11 pesticides and MPTP dysregulate 46 proteins associated with 31 molecular pathways in dopaminergic neurons. Dysregulation of these molecular pathways depicts a common mechanism between MTPT and pesticides that causes selective degeneration of dopamine neurons in PD pathogenesis. For instance, sphingosine-1-phosphate receptor 1 (S1P1) is one of the 46 proteins that play a crucial role in regulating inflammation. Notably, Pépin É et al., 2020, demonstrate the preventive role of oral S1P1 agonists against MPTP-induced mice model that protects against nigrostriatal neuronal loss and motor dysfunction [27]. Thus, the modulation and targeted therapy of S1P1 help protect neurons from MPTP neurotoxicity [28]. Likewise, Wnt signaling is one of the 31 molecular pathways identified in this study. Marchetti B, 2018 reports that exposure to MPTP induces microglial activation, which upregulates pro-inflammatory molecules including TNF-α, IL-1β, Wnt5a, iNOS, and reactive oxygen/nitrogen species [32]. Upregulation of inflammatory mechanisms disturbs Wnt/-catenin signaling in dopamine neurons, which are involved in neurogenesis [33]. Furthermore, dysregulation of Wnt/β-catenin signaling activates GSK-3β, which promotes β-catenin dependent neuron degradation and affects dopamine neuron vulnerability [34]. Similarly, involvement in mTOR signaling is noticed in this analysis. mTOR signaling plays a vital role in mitochondrial biogenesis at the transcriptional and post-translational levels [35,36]. Dysregulation of mTOR signaling is noticed in PD, which alters cellular bioenergetics and mitochondrial biogenesis [37,38].

On the other hand, among 46 crucial proteins, CTNNB1, NDUFS6, and CAV1 are top-ranked and chosen to be assessed for their expression in PD on pesticide exposure. All three top genes are well recognized for their involvement in neuronal function. CTNNB1 (beta-catenin 1) is an important modulator that regulates the Wnt signaling pathway. CTNNB1 activates Wnt signaling via calcium/calmodulin-dependent kinase II (CamKII). Moreover, beta-catenin1 regulates Jun-N-terminal kinase (JNK), leading to cell polarity (PCP) pathways [39]. Calcium ions act as secondary messengers for β-catenin-independent Wnt signaling [40,41,42]. Calcium plays a vital role in intracellular communication and polarizes the neurons [41]. Polarized neurons are more sensitive to Ca^2+^ influx and regulate neuronal functions and synaptic plasticity [42]. An increased cytosolic level of Ca^2+^ regulates diverse pathways associated with PD, including calmodulin (CaM). Additionally, PD-associated pesticides (such as paraquat, MPTP, and rotenone) inhibit the mitochondrial Ca^2+^ uniporter (MCU) complex and mitochondrial complex I, which increase cellular ROS and cause neuronal death [43,44,45].

Caveolin 1 (CAV1) is a membrane protein predominantly expressed in neurons involved in the aging process [46,47,48]. CAV1 interacts with the cytoskeleton, which promotes postsynaptic intracellular signaling [46,47,48]. Interestingly, increased CAV1 expression facilitates the uptake of α-syn into neurons, which forms Lewy body-like inclusion bodies that promote PD pathogenesis [46]. Moreover, CAV1 actively participates in learning, memory, drug addiction habits, and neuronal functional development [46,47,48]. A recent study by Li Y et al., 2021 demonstrates that the altered expression of the CAV1 protein promotes disruption in intracellular calcium homeostasis signaling pathways, which causes neurodegenerative diseases [49]. Our previous study demonstrated that the increased CSF calcium contributes to altered ion transport across the BBB and the blood–CSF barrier in PD [50]. Likewise, NDUFS6 (one of the top ranked proteins) is an accessory subunit associated with mitochondrial complex I involved in NADH dehydrogenase related respiratory chain processes in mitochondria [51]. NDUFS6 has a Zn-binding site that promotes the biogenesis of mitochondrial complex I [52,53]. Chinta SJ et al., 2011 show that the S-nitrosylation mechanism in mitochondrial complex I motif (NDUFS6) in mouse models and humans promotes the development of PD [54].

This current study provides several potential advantages by suggesting (1) how pesticides trigger downstream molecular mechanisms and activate pathogenic molecular pathways in PD; (2) the crucial role of these top genes in PD pathology associating pesticide exposure; and (3) the altered expression of these top-ranked genes (CTNNB1, NDUFS6, and CAV1) in PBMC results may pave the way for developing biomarkers that enable the screening of pesticide-associated PD. Alternatively, this study has a few limitations that need to be considered: (1) Assessment of limited participants for GC-MS pesticide assessment and gene expression analysis; (2) the immediate response of pesticides was assessed at the gene level, whereas protein expression was not established; (3) the recruited participants for this study belonged to a specific ethnicity (South Indian); and (4) the duration of exposure to pesticides was not assessed. Therefore, the current experimental investigations need to be continued with larger samples across various populations. However, at this juncture, this study has opened up potential candidate markers that may shed light on screening pesticide-exposed PD patients for better disease management.

## 5. Conclusions

In conclusion, this study combined computational and molecular approaches to investigate the molecular behavior of the eleven most common pesticides linked to Parkinson’s disease. Our analysis demonstrates a possible common mechanism between pesticides and the MPTP by activating critical pathogenic molecular pathways. Furthermore, this study shed light on the altered gene expression of CTNNB1, NDUFS6, and CAV1 in PBMC, which could serve as biomarkers for detecting pesticide-related Parkinson’s disease. More research with larger sample sizes across different ethnicities, as well as concurrent protein analysis, is needed to advance these identified markers closer to clinical application and management for Parkinson’s disease.

## Figures and Tables

**Table 1:** List of gene-specific primers.

GENE (*NCBI Accession*)	Forward Primer	Reverse Primer	Amplicon Size (Base Pair)
CTNNB1 (*XM_054345317*)	CACAAGCAGAGTGCTGAAGGTG	GATTCCTGAGAGTCCAAAGACAG	146
NDUFS6 (*NM_004553*)	GTTCAGACAGCACCACCACT	CACCAGGAATACCCTTCGCA	124
CAV1 (*NM_001172897*)	AAGGGACACACAGTTTTGACG	TTGGCACCAGGAAAATTAAAA	372
GAPDH (*NM_001289745*)	AAGGTGAAGGTCGGAGTCAA	ACATGTAAACCATGTAGTTGAGGT	133

**Table 2:** List of 46 proteins activated upon pesticide toxicity.

Gene Symbol	Gene Name Expansion
ABL1	ABL proto-oncogene 1, non-receptor tyrosine kinase
ACVR1B	Activin a receptor type 1b
APP	Amyloid beta precursor protein
ARAP1	ArfGAP with RhoGAP domain, ankyrin repeat and PH domain 1
BAG6	BAG cochaperone 6
CAV1	Caveolin 1
CDC37	Cell division cycle 37, HSP90 cochaperone
CDC42	Cell division cycle 42
CDK14	Cyclin dependent kinase 14
CHGB	ChromograninB
CHN1	Chimerin 1
CSNK1A1	Casein kinase 1 alpha 1
CTNNB1	Catenin beta 1
CTSK	CathepsinK
EP300	E1A binding protein p300
FGF1	Fibroblast growth factor 1
FGF18	Fibroblast growth factor 18
FGF5	Fibroblast growth factor 5
FGFR2	Fibroblast growth factor receptor 2
GTF3C1	General transcription factor IIIC subunit 1
HNRNPL	Heterogeneous nuclear ribonucleoprotein L
IGSF1	Immunoglobulin superfamily member 1
IKBKB	Inhibitor of nuclear factor kappa B kinase subunit beta
INHBA	Inhibinsubunit beta A
LGALS8	Galectin 8
LMO3	LIM domain only 3
MAP3K7	Mitogen-activated protein kinase kinasekinase 7
NCOA1	Nuclear receptor coactivator 1
NDUFS6	NADH:ubiquinoneoxidoreductase subunit S6
NFKB1	Nuclear factor kappa B subunit 1
PEG10	Paternally expressed 10
PLEKHB1	Pleckstrinhomology domain containing B1
PPP3CC	Protein phosphatase 3 catalytic subunit gamma
PTBP1	Polypyrimidine tract binding protein 1
RAB6B	RAB6B, member RAS oncogene family
RAN	RAN, member RAS oncogene family
RASL12	RAS like family 12
SH2B1	SH2B adaptor protein 1
SHC1	SHC adaptor protein 1
GUSBP14	GUSB pseudogene 14
SMAD2	SMAD family member 2
SRPK2	SRSF protein kinase 2
STAT1	Signal transducer and activator of transcription 1
STAT3	Signal transducer and activator of transcription 3
YWHAZ	Tyrosine 3-monooxygenase/tryptophan 5-monooxygenase activation protein zeta
ZXDC	ZXD family zinc finger C

**Table 3:** Molecular pathways associated with 46 common proteins from 11 pesticides and MPTP.

Sl.No	Molecular Pathway
1	Sphingolipid signaling pathway
2	Phospholipase D signaling pathway
3	mTOR signaling pathway
4	AMPK signaling pathway
5	Longevity regulating pathway
6	Notch signaling pathway
7	Hedgehog signaling pathway
8	Apelin signaling pathway
9	GnRH signaling pathway
10	Oxytocin signaling pathway
11	ErbB signaling pathway
12	cAMP signaling pathway
13	VEGF signaling pathway
14	Insulin signaling pathway
15	Estrogen signaling pathway
16	Glucagon signaling pathway
17	FoxO signaling pathway
18	B cell receptor signaling pathway
19	TNF signaling pathway
20	Adipocytokine signaling pathway
21	Relaxin signaling pathway
22	Prolactin signaling pathway
23	Thyroid hormone signaling pathway
24	Calcium signaling pathway
25	Wnt signaling pathway
26	T cell receptor signaling pathway
27	Rap1 signaling pathway
28	Chemokine signaling pathway
29	Neurotrophin signaling pathway
30	MAPK signaling pathway
31	Ras signaling pathway

**Table 4:** Demographic details of participants selected for the study.

Clinical Parameters	Pesticide Exposed PD (Group-A: n = 23)	Non-Pesticide Exposed PD (Group-B; n = 25)	Healthy Control (Group-C: n = 21)	Statistical Significance		
				(Group-A Vs. Group-B)	(Group-A Vs. Group-C)	(Group-B Vs. Group-C)
Age (Years)	57 ± 9.8	52 ± 4.8	53 ± 6.2	X	X	X
Gender (M: male, F: female)	M:13; F: 10	M: 15; F: 10	M: 11; F: 10	--	--	--
Total for parts I–III (items 1–31)	31.6 ± 2.4	30.2 ± 3.1		X	--	--
Motor Scale (items 18–31)	12.9 ±4.1	11.8± 5.7		X	--	--
Hoehn and Yahr stage	2.4 ±1.9	2.8± 1.2		X	--	--

X: no statistical significance; --: not assessed.

## Data Availability

Data are available on request.

## Supplementary Materials

The following supporting information can be downloaded at: https://www.mdpi.com/article/10.3390/brainsci13071003/s1, Supplementary File S1: Proteins involved in the MPTP network.
