# Supplementary material for: Eleven Crucial Pesticides Appear to Regulate Key Genes That Link MPTP Mechanism to Cause Parkinson’s Disease through the Selective Degeneration of Dopamine Neurons
Source: Brain Sci. 2023 Jun 28;13(7):1003. doi: 10.3390/brainsci13071003 (PMC10377611; doi:10.3390/brainsci13071003)
Supplement: Supplementary file 1 [file brainsci-13-01003-s001.zip › brainsci-2458123-supplementary.pdf]

Supplementary File S1: Proteins involved in the MPTP network.

Entrez ID	Gene symbol	Description
10347	ABCA7	ATP-binding cassette, sub-family A (ABC1), member 7
10058	ABCB6	ATP-binding cassette, sub-family B (MDR/TAP), member 6 (Langereis blood group)
25	ABL1	ABL proto-oncogene 1, non-receptor tyrosine kinase
28976	ACAD9	acyl-CoA dehydrogenase family, member 9
9744	ACAP1	ArfGAP with coiled-coil, ankyrin repeat and PH domains 1
53	ACP2	acid phosphatase 2, lysosomal
60	ACTB	actin, beta
90	ACVR1	activin A receptor, type I
91	ACVR1B	activin A receptor, type IB
130399	ACVR1C	activin A receptor, type IC
92	ACVR2A	activin A receptor, type IIA
93	ACVR2B	activin A receptor, type IIB
94	ACVRL1	activin A receptor type II-like 1
102	ADAM10	ADAM metallopeptidase domain 10
6868	ADAM17	ADAM metallopeptidase domain 17
8754	ADAM9	ADAM metallopeptidase domain 9
119	ADD2	adducin 2 (beta)
22859	ADGRL1	adhesion G protein-coupled receptor L1
134	ADORA1	adenosine A1 receptor
154	ADRB2	adrenoceptor beta 2, surface
156	ADRBK1	adrenergic, beta, receptor kinase 1
27161	AGO2	argonaute RISC catalytic component 2
10598	AHSA1	AHA1, activator of heat shock 90kDa protein ATPase homolog 1 (yeast)
207	AKT1	v-akt murine thymoma viral oncogene homolog 1
213	ALB	albumin

221	ALDH3B1	aldehyde dehydrogenase 3 family, member B1
84920	ALG10	ALG10, alpha-1,2-glucosyltransferase
238	ALK	anaplastic lymphoma receptor tyrosine kinase
239	ALOX12	arachidonate 12-lipoxygenase
269	AMHR2	anti-Mullerian hormone receptor, type II
51433	ANAPC5	anaphase promoting complex subunit 5
284	ANGPT1	angiopoietin 1
23452	ANGPTL2	angiopoietin-like 2
27329	ANGPTL3	angiopoietin-like 3
253935	ANGPTL5	angiopoietin-like 5
286	ANK1	ankyrin 1, erythrocytic
56899	ANKS1B	ankyrin repeat and sterile alpha motif domain containing 1B
8907	AP1M1	adaptor-related protein complex 1, mu 1 subunit
163	AP2B1	adaptor-related protein complex 2, beta 1 subunit
1173	AP2M1	adaptor-related protein complex 2, mu 1 subunit
323	APBB2	amyloid beta (A4) precursor protein-binding, family B, member 2
325	APCS	amyloid P component, serum
338	APOB	apolipoprotein B
351	APP	amyloid beta (A4) precursor protein
26060	APPL1	adaptor protein, phosphotyrosine interaction, PH domain and leucine zipper containing 1
54840	APTX	aprataxin
116985	ARAP1	ArfGAP with RhoGAP domain, ankyrin repeat and PH domain 1
55738	ARFGAP1	ADP-ribosylation factor GTPase activating protein 1
55843	ARHGAP15	Rho GTPase activating protein 15
57514	ARHGAP31	Rho GTPase activating protein 31
9743	ARHGAP32	Rho GTPase activating protein 32
2909	ARHGAP35	Rho GTPase activating protein 35
394	ARHGAP5	Rho GTPase activating protein 5
9459	ARHGEF6	Rac/Cdc42 guanine nucleotide exchange factor (GEF) 6
285598	ARL10	ADP-ribosylation factor-like 10

379	ARL4D	ADP-ribosylation factor-like 4D
10550	ARL6IP5	ADP-ribosylation factor-like 6 interacting protein 5
55207	ARL8B	ADP-ribosylation factor-like 8B
64860	ARMCX5	armadillo repeat containing, X-linked 5
405	ARNT	aryl hydrocarbon receptor nuclear translocator
409	ARRB2	arrestin, beta 2
57561	ARRDC3	arrestin domain containing 3
51666	ASB4	ankyrin repeat and SOCS box containing 4
9070	ASH2L	ash2 (absent, small, or homeotic)-like (Drosophila)
445	ASS1	argininosuccinate synthase 1
1386	ATF2	activating transcription factor 2
467	ATF3	activating transcription factor 3
476	ATP1A1	ATPase, Na+/K+ transporting, alpha 1 polypeptide
483	ATP1B3	ATPase, Na+/K+ transporting, beta 3 polypeptide
488	ATP2A2	ATPase, Ca++ transporting, cardiac muscle, slow twitch 2
506	ATP5B	ATP synthase, H+ transporting, mitochondrial F1 complex, beta polypeptide
515	ATP5F1	ATP synthase, H+ transporting, mitochondrial Fo complex, subunit B1
10159	ATP6AP2	ATPase, H+ transporting, lysosomal accessory protein 2
525	ATP6V1B1	ATPase, H+ transporting, lysosomal 56/58kDa, V1 subunit B1
6310	ATXN1	ataxin 1
550	AUP1	ancient ubiquitous protein 1
6790	AURKA	aurora kinase A
9212	AURKB	aurora kinase B
551	AVP	arginine vasopressin
552	AVPR1A	arginine vasopressin receptor 1A
553	AVPR1B	arginine vasopressin receptor 1B
554	AVPR2	arginine vasopressin receptor 2
558	AXL	AXL receptor tyrosine kinase
567	B2M	beta-2-microglobulin
2683	B4GALT1	UDP-Gal:betaGlcNAc beta 1,4- galactosyltransferase, polypeptide 1

9334	B4GALT5	UDP-Gal:betaGlcNAc beta 1,4- galactosyltransferase, polypeptide 5
572	BAD	BCL2-associated agonist of cell death
9530	BAG4	BCL2-associated athanogene 4
7917	BAG6	BCL2-associated athanogene 6
55971	BAIAP2L1	BAI1-associated protein 2-like 1
25805	BAMBI	BMP and activin membrane-bound inhibitor
622	BDH1	3-hydroxybutyrate dehydrogenase, type 1
627	BDNF	brain-derived neurotrophic factor
633	BGN	biglycan
79365	BHLHE41	basic helix-loop-helix family, member e41
55909	BIN3	bridging integrator 3
329	BIRC2	baculoviral IAP repeat containing 2
642	BLMH	bleomycin hydrolase
9210	BMP15	bone morphogenetic protein 15
650	BMP2	bone morphogenetic protein 2
652	BMP4	bone morphogenetic protein 4
654	BMP6	bone morphogenetic protein 6
655	BMP7	bone morphogenetic protein 7
657	BMPR1A	bone morphogenetic protein receptor, type IA
658	BMPR1B	bone morphogenetic protein receptor, type IB
659	BMPR2	bone morphogenetic protein receptor, type II (serine/threonine kinase)
79866	BORA	bora, aurora kinase A activator
140707	BRI3BP	BRI3 binding protein
55299	BRIX1	BRX1, biogenesis of ribosomes
25855	BRMS1	breast cancer metastasis suppressor 1
684	BST2	bone marrow stromal cell antigen 2
55643	BTBD2	BTB (POZ) domain containing 2
8945	BTRC	beta-transducin repeat containing E3 ubiquitin protein ligase
148223	C19orf25	chromosome 19 open reading frame 25
9847	C2CD5	C2 calcium-dependent domain containing 5

205327	C2orf69	chromosome 2 open reading frame 69
57827	C6orf47	chromosome 6 open reading frame 47
23705	CADM1	cell adhesion molecule 1
796	CALCA	calcitonin-related polypeptide alpha
799	CALCR	calcitonin receptor
801	CALM1	calmodulin 1 (phosphorylase kinase, delta)
805	CALM2	calmodulin 2 (phosphorylase kinase, delta)
808	CALM3	calmodulin 3 (phosphorylase kinase, delta)
55832	CAND1	cullin-associated and neddylation-dissociated 1
826	CAPNS1	calpain, small subunit 1
833	CARS	cysteinyl-tRNA synthetase
8573	CASK	calcium/calmodulin-dependent serine protein kinase (MAGUK family)
841	CASP8	caspase 8, apoptosis-related cysteine peptidase
857	CAV1	caveolin 1, caveolae protein, 22kDa
859	CAV3	caveolin 3
867	CBL	Cbl proto-oncogene, E3 ubiquitin protein ligase
147872	CCDC155	coiled-coil domain containing 155
149483	CCDC17	coiled-coil domain containing 17
100133941	CD24	CD24 molecule
919	CD247	CD247 molecule
948	CD36	CD36 molecule (thrombospondin receptor)
952	CD38	CD38 molecule
920	CD4	CD4 molecule
960	CD44	CD44 molecule (Indian blood group)
928	CD9	CD9 molecule
991	CDC20	cell division cycle 20
11140	CDC37	cell division cycle 37
998	CDC42	cell division cycle 42
23580	CDC42EP4	CDC42 effector protein (Rho GTPase binding) 4
983	CDK1	cyclin-dependent kinase 1

5218	CDK14	cyclin-dependent kinase 14
5128	CDK17	cyclin-dependent kinase 17
1017	CDK2	cyclin-dependent kinase 2
1019	CDK4	cyclin-dependent kinase 4
1021	CDK6	cyclin-dependent kinase 6
8814	CDKL1	cyclin-dependent kinase-like 1 (CDC2-related kinase)
284040	CDRT4	CMT1A duplicated region transcript 4
9425	CDYL	chromodomain protein, Y-like
1051	CEBPB	CCAAT/enhancer binding protein (C/EBP), beta
1952	CELSR2	cadherin, EGF LAG seven-pass G-type receptor 2
201161	CENPV	centromere protein V
57562	CEP126	centrosomal protein 126kDa
91012	CERS5	ceramide synthase 5
253782	CERS6	ceramide synthase 6
1080	CFTR	cystic fibrosis transmembrane conductance regulator (ATP-binding cassette sub-family C, member 7)
1114	CHGB	chromogranin B
1123	CHN1	chimerin 1
1124	CHN2	chimerin 2
8646	CHRD	chordin
1147	CHUK	conserved helix-loop-helix ubiquitous kinase
1179	CLCA1	chloride channel accessory 1
9635	CLCA2	chloride channel accessory 2
64581	CLEC7A	C-type lectin domain family 7, member A
1195	CLK1	CDC-like kinase 1
1201	CLN3	ceroid-lipofuscinosis, neuronal 3
1191	CLU	clusterin
54918	CMTM6	CKLF-like MARVEL transmembrane domain containing 6
1268	CNR1	cannabinoid receptor 1 (brain)
1270	CNTF	ciliary neurotrophic factor
1272	CNTN1	contactin 1

22837	COBLL1	cordon-bleu WH2 repeat protein-like 1
1308	COL17A1	collagen, type XVII, alpha 1
80781	COL18A1	collagen, type XVIII, alpha 1
1277	COL1A1	collagen, type I, alpha 1
1278	COL1A2	collagen, type I, alpha 2
1314	COPA	coatomer protein complex, subunit alpha
10987	COPS5	COP9 signalosome subunit 5
10980	COPS6	COP9 signalosome subunit 6
1362	CPD	carboxypeptidase D
78987	CRELD1	cysteine-rich with EGF-like domains 1
1392	CRH	corticotropin releasing hormone
1395	CRHR2	corticotropin releasing hormone receptor 2
1398	CRK	v-crk avian sarcoma virus CT10 oncogene homolog
1399	CRKL	v-crk avian sarcoma virus CT10 oncogene homolog-like
1410	CRYAB	crystallin, alpha B
1435	CSF1	colony stimulating factor 1 (macrophage)
1445	CSK	c-src tyrosine kinase
1452	CSNK1A1	casein kinase 1, alpha 1
1454	CSNK1E	casein kinase 1, epsilon
1455	CSNK1G2	casein kinase 1, gamma 2
1459	CSNK2A2	casein kinase 2, alpha prime polypeptide
1464	CSPG4	chondroitin sulfate proteoglycan 4
1490	CTGF	connective tissue growth factor
1499	CTNNB1	catenin (cadherin-associated protein), beta 1, 88kDa
1513	CTSK	cathepsin K
8454	CUL1	cullin 1
8452	CUL3	cullin 3
8065	CUL5	cullin 5
9820	CUL7	cullin 7
1540	CYLD	cylindromatosis (turban tumor syndrome)

1601	DAB2	Dab, mitogen-responsive phosphoprotein, homolog 2 (Drosophila)
1605	DAG1	dystroglycan 1 (dystrophin-associated glycoprotein 1)
1612	DAPK1	death-associated protein kinase 1
23604	DAPK2	death-associated protein kinase 2
25853	DCAF12	DDB1 and CUL4 associated factor 12
55827	DCAF6	DDB1 and CUL4 associated factor 6
10238	DCAF7	DDB1 and CUL4 associated factor 7
1634	DCN	decorin
1642	DDB1	damage-specific DNA binding protein 1, 127kDa
11269	DDX19B	DEAD (Asp-Glu-Ala-Asp) box polypeptide 19B
11218	DDX20	DEAD (Asp-Glu-Ala-Asp) box polypeptide 20
57062	DDX24	DEAD (Asp-Glu-Ala-Asp) box helicase 24
317781	DDX51	DEAD (Asp-Glu-Ala-Asp) box polypeptide 51
55601	DDX60	DEAD (Asp-Glu-Ala-Asp) box polypeptide 60
23405	DICER1	dicer 1, ribonuclease type III
22894	DIS3	DIS3 homolog, exosome endoribonuclease and 3'-5' exoribonuclease
1738	DLD	dihydrolipoamide dehydrogenase
1739	DLG1	discs, large homolog 1 (Drosophila)
1740	DLG2	discs, large homolog 2 (Drosophila)
1742	DLG4	discs, large homolog 4 (Drosophila)
9231	DLG5	discs, large homolog 5 (Drosophila)
8788	DLK1	delta-like 1 homolog (Drosophila)
1746	DLX2	distal-less homeobox 2
55172	DNAAF2	dynein, axonemal, assembly factor 2
55466	DNAJA4	DnaJ (Hsp40) homolog, subfamily A, member 4
1803	DPP4	dipeptidyl-peptidase 4
1829	DSG2	desmoglein 2
667	DST	dystonin
54808	DYM	dymeclin
8444	DYRK3	dual-specificity tyrosine-(Y)-phosphorylation regulated kinase 3

10682	EBP	emopamil binding protein (sterol isomerase)
51295	ECSIT	ECSIT signalling integrator
8726	EED	embryonic ectoderm development
1942	EFNA1	ephrin-A1
1948	EFNB2	ephrin-B2
1949	EFNB3	ephrin-B3
1956	EGFR	epidermal growth factor receptor
440275	EIF2AK4	eukaryotic translation initiation factor 2 alpha kinase 4
1967	EIF2B1	eukaryotic translation initiation factor 2B, subunit 1 alpha, 26kDa
8665	EIF3F	eukaryotic translation initiation factor 3, subunit F
1978	EIF4EBP1	eukaryotic translation initiation factor 4E binding protein 1
3692	EIF6	eukaryotic translation initiation factor 6
146956	EME1	essential meiotic structure-specific endonuclease 1
2014	EMP3	epithelial membrane protein 3
8507	ENC1	ectodermal-neural cortex 1 (with BTB domain)
2022	ENG	endoglin
5168	ENPP2	ectonucleotide pyrophosphatase/phosphodiesterase 2
2033	EP300	E1A binding protein p300
2041	EPHA1	EPH receptor A1
1969	EPHA2	EPH receptor A2
2043	EPHA4	EPH receptor A4
29924	EPN1	epsin 1
2060	EPS15	epidermal growth factor receptor pathway substrate 15
51752	ERAP1	endoplasmic reticulum aminopeptidase 1
2064	ERBB2	erb-b2 receptor tyrosine kinase 2
285141	ERICH2	glutamate-rich 2
54206	ERRFI1	ERBB receptor feedback inhibitor 1
2099	ESR1	estrogen receptor 1
2100	ESR2	estrogen receptor 2 (ER beta)
2103	ESRRB	estrogen-related receptor beta

59271	EVA1C	eva-1 homolog C (C. elegans)
2130	EWSR1	EWS RNA-binding protein 1
2146	EZH2	enhancer of zeste 2 polycomb repressive complex 2 subunit
7430	EZR	ezrin
2170	FABP3	fatty acid binding protein 3, muscle and heart
8772	FADD	Fas (TNFRSF6)-associated via death domain
23197	FAF2	Fas associated factor family member 2
143684	FAM76B	family with sequence similarity 76, member B
55120	FANCL	Fanconi anemia, complementation group L
57697	FANCM	Fanconi anemia, complementation group M
2191	FAP	fibroblast activation protein, alpha
10160	FARP1	FERM, RhoGEF (ARHGEF) and pleckstrin domain protein 1 (chondrocyte-derived)
355	FAS	Fas cell surface death receptor
54850	FBXL12	F-box and leucine-rich repeat protein 12
54620	FBXL19	F-box and leucine-rich repeat protein 19
26260	FBXO25	F-box protein 25
55030	FBXO34	F-box protein 34
26270	FBXO6	F-box protein 6
23291	FBXW11	F-box and WD repeat domain containing 11
2207	FCER1G	Fc fragment of IgE, high affinity I, receptor for; gamma polypeptide
2209	FCGR1A	Fc fragment of IgG, high affinity Ia, receptor (CD64)
2214	FCGR3A	Fc fragment of IgG, low affinity IIIa, receptor (CD16a)
2215	FCGR3B	Fc fragment of IgG, low affinity IIIb, receptor (CD16b)
2246	FGF1	fibroblast growth factor 1 (acidic)
8822	FGF17	fibroblast growth factor 17
8817	FGF18	fibroblast growth factor 18
2247	FGF2	fibroblast growth factor 2 (basic)
8074	FGF23	fibroblast growth factor 23
2248	FGF3	fibroblast growth factor 3
2249	FGF4	fibroblast growth factor 4

2250	FGF5	fibroblast growth factor 5
2251	FGF6	fibroblast growth factor 6
2252	FGF7	fibroblast growth factor 7
2253	FGF8	fibroblast growth factor 8 (androgen-induced)
2254	FGF9	fibroblast growth factor 9
2263	FGFR2	fibroblast growth factor receptor 2
2261	FGFR3	fibroblast growth factor receptor 3
2267	FGL1	fibrinogen-like 1
2280	FKBP1A	FK506 binding protein 1A, 12kDa
2316	FLNA	filamin A, alpha
10211	FLOT1	flotillin 1
2324	FLT4	fms-related tyrosine kinase 4
2335	FN1	fibronectin 1
22862	FNDC3A	fibronectin type III domain containing 3A
2339	FNTA	farnesyltransferase, CAAX box, alpha
2348	FOLR1	folate receptor 1
2350	FOLR2	folate receptor Beta
2355	FOSL2	FOS-like antigen 2
399823	FOXI2	forkhead box I2
50943	FOXP3	forkhead box P3
283150	FOXR1	forkhead box R1
341640	FREM2	FRAS1 related extracellular matrix protein 2
2495	FTH1	ferritin, heavy polypeptide 1
8880	FUBP1	far upstream element (FUSE) binding protein 1
5045	FURIN	furin (paired basic amino acid cleaving enzyme)
2526	FUT4	fucosyltransferase 4 (alpha (1,3) fucosyltransferase, myeloid-specific)
2534	FYN	FYN proto-oncogene, Src family tyrosine kinase
8324	FZD7	frizzled class receptor 7
11337	GABARAP	GABA(A) receptor-associated protein
2597	GAPDH	glyceraldehyde-3-phosphate dehydrogenase

2621	GAS6	growth arrest-specific 6
8200	GDF5	growth differentiation factor 5
392255	GDF6	growth differentiation factor 6
2661	GDF9	growth differentiation factor 9
50628	GEMIN4	gem (nuclear organelle) associated protein 4
79833	GEMIN6	gem (nuclear organelle) associated protein 6
79760	GEMIN7	gem (nuclear organelle) associated protein 7
54960	GEMIN8	gem (nuclear organelle) associated protein 8
2670	GFAP	glial fibrillary acidic protein
26088	GGA1	golgi-associated, gamma adaptin ear containing, ARF binding protein 1
23062	GGA2	golgi-associated, gamma adaptin ear containing, ARF binding protein 2
23163	GGA3	golgi-associated, gamma adaptin ear containing, ARF binding protein 3
10755	GIPC1	GIPC PDZ domain containing family, member 1
28964	GIT1	G protein-coupled receptor kinase interacting ArfGAP 1
2706	GJB2	gap junction protein, beta 2, 26kDa
2710	GK	glycerol kinase
2744	GLS	glutaminase
2752	GLUL	glutamate-ammonia ligase
2767	GNA11	guanine nucleotide binding protein (G protein), alpha 11 (Gq class)
10672	GNA13	guanine nucleotide binding protein (G protein), alpha 13
2769	GNA15	guanine nucleotide binding protein (G protein), alpha 15 (Gq class)
2770	GNAI1	guanine nucleotide binding protein (G protein), alpha inhibiting activity polypeptide 1
2771	GNAI2	guanine nucleotide binding protein (G protein), alpha inhibiting activity polypeptide 2
2776	GNAQ	guanine nucleotide binding protein (G protein), q polypeptide
2783	GNB2	guanine nucleotide binding protein (G protein), beta polypeptide 2
2784	GNB3	guanine nucleotide binding protein (G protein), beta polypeptide 3
2802	GOLGA3	golgin A3
9950	GOLGA5	golgin A5
64083	GOLPH3	golgi phosphoprotein 3 (coat-protein)
51026	GOLT1B	golgi transport 1B

2811	GP1BA	glycoprotein Ib (platelet), alpha polypeptide
51206	GP6	glycoprotein VI (platelet)
65056	GPBP1	GC-rich promoter binding protein 1
653519	GPR89A	G protein-coupled receptor 89A
51463	GPR89B	G protein-coupled receptor 89B
9737	GPRASP1	G protein-coupled receptor associated sorting protein 1
114928	GPRASP2	G protein-coupled receptor associated sorting protein 2
63940	GPSM3	G-protein signaling modulator 3
57655	GRAMD1A	GRAM domain containing 1A
160622	GRASP	GRP1 (general receptor for phosphoinositides 1)-associated scaffold protein
2888	GRB14	growth factor receptor-bound protein 14
2885	GRB2	growth factor receptor-bound protein 2
23426	GRIP1	glutamate receptor interacting protein 1
2870	GRK6	G protein-coupled receptor kinase 6
2911	GRM1	glutamate receptor, metabotropic 1
2912	GRM2	glutamate receptor, metabotropic 2
2918	GRM8	glutamate receptor, metabotropic 8
2896	GRN	granulin
2936	GSR	glutathione reductase
9569	GTF2IRD1	GTF2I repeat domain containing 1
2975	GTF3C1	general transcription factor IIIC, polypeptide 1, alpha 220kDa
60558	GUF1	GUF1 homolog, GTPase
3020	H3F3A	H3 histone, family 3A
3021	H3F3B	H3 histone, family 3B (H3.3B)
3050	HBZ	hemoglobin, zeta
10870	HCST	hematopoietic cell signal transducer
10014	HDAC5	histone deacetylase 5
253012	HEPACAM2	HEPACAM family member 2
3077	HFE	hemochromatosis
9146	HGS	hepatocyte growth factor-regulated tyrosine kinase substrate

3024	HIST1H1A	histone cluster 1, H1a
3009	HIST1H1B	histone cluster 1, H1b
3006	HIST1H1C	histone cluster 1, H1c
3007	HIST1H1D	histone cluster 1, H1d
3008	HIST1H1E	histone cluster 1, H1e
3010	HIST1H1T	histone cluster 1, H1t
3106	HLA-B	major histocompatibility complex, class I, B
3107	HLA-C	major histocompatibility complex, class I, C
3113	HLA-DPA1	major histocompatibility complex, class II, DP alpha 1
3134	HLA-F	major histocompatibility complex, class I, F
3135	HLA-G	major histocompatibility complex, class I, G
3146	HMGB1	high mobility group box 1
3161	HMMR	hyaluronan-mediated motility receptor (RHAMM)
3184	HNRNPD	heterogeneous nuclear ribonucleoprotein D (AU-rich element RNA binding protein 1, 37kDa)
3191	HNRNPL	heterogeneous nuclear ribonucleoprotein L
3192	HNRNPU	heterogeneous nuclear ribonucleoprotein U (scaffold attachment factor A)
9456	HOMER1	homer scaffolding protein 1
9455	HOMER2	homer scaffolding protein 2
3240	HP	haptoglobin
90161	HS6ST2	heparan sulfate 6-O-sulfotransferase 2
3290	HSD11B1	hydroxysteroid (11-beta) dehydrogenase 1
3295	HSD17B4	hydroxysteroid (17-beta) dehydrogenase 4
3320	HSP90AA1	heat shock protein 90kDa alpha (cytosolic), class A member 1
3326	HSP90AB1	heat shock protein 90kDa alpha (cytosolic), class B member 1
259217	HSPA12A	heat shock 70kDa protein 12A
6782	HSPA13	heat shock protein 70kDa family, member 13
3303	HSPA1A	heat shock 70kDa protein 1A
3304	HSPA1B	heat shock 70kDa protein 1B
3308	HSPA4	heat shock 70kDa protein 4
3312	HSPA8	heat shock 70kDa protein 8

3356	HTR2A	5-hydroxytryptamine (serotonin) receptor 2A, G protein-coupled
3064	HTT	huntingtin
10075	HUWE1	HECT, UBA and WWE domain containing 1, E3 ubiquitin protein ligase
3383	ICAM1	intercellular adhesion molecule 1
7087	ICAM5	intercellular adhesion molecule 5, telencephalin
79664	ICE2	interactor of little elongation complex ELL subunit 2
29851	ICOS	inducible T-cell co-stimulator
3458	IFNG	interferon, gamma
3547	IGSF1	immunoglobulin superfamily, member 1
3551	IKBKB	inhibitor of kappa light polypeptide gene enhancer in B-cells, kinase beta
9641	IKBKE	inhibitor of kappa light polypeptide gene enhancer in B-cells, kinase epsilon
8517	IKBKG	inhibitor of kappa light polypeptide gene enhancer in B-cells, kinase gamma
3596	IL13	interleukin 13
3597	IL13RA1	interleukin 13 receptor, alpha 1
3601	IL15RA	interleukin 15 receptor, alpha
3554	IL1R1	interleukin 1 receptor, type I
9173	IL1RL1	interleukin 1 receptor-like 1
3558	IL2	interleukin 2
3559	IL2RA	interleukin 2 receptor, alpha
3560	IL2RB	interleukin 2 receptor, beta
3561	IL2RG	interleukin 2 receptor, gamma
90865	IL33	interleukin 33
3565	IL4	interleukin 4
3566	IL4R	interleukin 4 receptor
3569	IL6	interleukin 6
3570	IL6R	interleukin 6 receptor
3572	IL6ST	interleukin 6 signal transducer
3611	ILK	integrin-linked kinase
80895	ILKAP	integrin-linked kinase-associated serine/threonine phosphatase
3615	IMPDH2	IMP (inosine 5'-monophosphate) dehydrogenase 2

3624	INHBA	inhibin, beta A
3625	INHBB	inhibin, beta B
3626	INHBC	inhibin, beta C
3636	INPPL1	inositol polyphosphate phosphatase-like 1
80789	INTS5	integrator complex subunit 5
51194	IPO11	importin 11
55705	IPO9	importin 9
3654	IRAK1	interleukin-1 receptor-associated kinase 1
3656	IRAK2	interleukin-1 receptor-associated kinase 2
11213	IRAK3	interleukin-1 receptor-associated kinase 3
51135	IRAK4	interleukin-1 receptor-associated kinase 4
3661	IRF3	interferon regulatory factor 3
83737	ITCH	itchy E3 ubiquitin protein ligase
3674	ITGA2B	integrin, alpha 2b (platelet glycoprotein IIb of IIb/IIIa complex, antigen CD41)
3675	ITGA3	integrin, alpha 3 (antigen CD49C, alpha 3 subunit of VLA-3 receptor)
3676	ITGA4	integrin, alpha 4 (antigen CD49D, alpha 4 subunit of VLA-4 receptor)
3678	ITGA5	integrin, alpha 5 (fibronectin receptor, alpha polypeptide)
3655	ITGA6	integrin, alpha 6
3685	ITGAV	integrin, alpha V
3688	ITGB1	integrin, beta 1 (fibronectin receptor, beta polypeptide, antigen CD29 includes MDF2, MSK12)
3690	ITGB3	integrin, beta 3 (platelet glycoprotein IIIa, antigen CD61)
3691	ITGB4	integrin, beta 4
3693	ITGB5	integrin, beta 5
3698	ITIH2	inter-alpha-trypsin inhibitor heavy chain 2
3702	ITK	IL2-inducible T-cell kinase
3708	ITPR1	inositol 1,4,5-trisphosphate receptor, type 1
3709	ITPR2	inositol 1,4,5-trisphosphate receptor, type 2
3716	JAK1	Janus kinase 1
3717	JAK2	Janus kinase 2
3728	JUP	junction plakoglobin

10300	KATNB1	katanin p80 (WD repeat containing) subunit B 1
3738	KCNA3	potassium channel, voltage gated shaker related subfamily A, member 3
3741	KCNA5	potassium channel, voltage gated shaker related subfamily A, member 5
338567	KCNK18	potassium channel, two pore domain subfamily K, member 18
79932	KIAA0319L	KIAA0319-like
57179	KIAA1191	KIAA1191
90231	KIAA2013	KIAA2013
3832	KIF11	kinesin family member 11
3805	KIR2DL4	killer cell immunoglobulin-like receptor, two domains, long cytoplasmic tail, 4
3815	KIT	v-kit Hardy-Zuckerman 4 feline sarcoma viral oncogene homolog
3831	KLC1	kinesin light chain 1
147700	KLC3	kinesin light chain 3
23588	KLHDC2	kelch domain containing 2
57626	KLHL1	kelch-like family member 1
283212	KLHL35	kelch-like family member 35
100528032	KLRC4-KLRK1	KLRC4-KLRK1 readthrough
10219	KLRG1	killer cell lectin-like receptor subfamily G, member 1
22914	KLRK1	killer cell lectin-like receptor subfamily K, member 1
4297	KMT2A	lysine (K)-specific methyltransferase 2A
3836	KPNA1	karyopherin alpha 1 (importin alpha 5)
3839	KPNA3	karyopherin alpha 3 (importin alpha 4)
3881	KRT31	keratin 31, type I
9119	KRT75	keratin 75, type II
3856	KRT8	keratin 8, type II
386681	KRTAP10-8	keratin associated protein 10-8
3897	L1CAM	L1 cell adhesion molecule
3920	LAMP2	lysosomal-associated membrane protein 2
10542	LAMTOR5	late endosomal/lysosomal adaptor, MAPK and MTOR activator 5
9741	LAPTM4A	lysosomal protein transmembrane 4 alpha
55353	LAPTM4B	lysosomal protein transmembrane 4 beta

3932	LCK	LCK proto-oncogene, Src family tyrosine kinase
3949	LDLR	low density lipoprotein receptor
221496	LEMD2	LEM domain containing 2
23484	LEPROTL1	leptin receptor overlapping transcript-like 1
3958	LGALS3	lectin, galactoside-binding, soluble, 3
3959	LGALS3BP	lectin, galactoside-binding, soluble, 3 binding protein
3964	LGALS8	lectin, galactoside-binding, soluble, 8
3965	LGALS9	lectin, galactoside-binding, soluble, 9
55366	LGR4	leucine-rich repeat containing G protein-coupled receptor 4
11024	LILRA1	leukocyte immunoglobulin-like receptor, subfamily A (with TM domain), member 1
11026	LILRA3	leukocyte immunoglobulin-like receptor, subfamily A (without TM domain), member 3
10859	LILRB1	leukocyte immunoglobulin-like receptor, subfamily B (with TM and ITIM domains), member 1
10288	LILRB2	leukocyte immunoglobulin-like receptor, subfamily B (with TM and ITIM domains), member 2
3987	LIMS1	LIM and senescent cell antigen-like domains 1
132660	LIN54	lin-54 DREAM MuvB core complex component
84894	LINGO1	leucine rich repeat and Ig domain containing 1
64327	LMBR1	limb development membrane protein 1
55885	LMO3	LIM domain only 3 (rhombotin-like 2)
22853	LMTK2	lemur tyrosine kinase 2
4012	LNPEP	leucyl/cystinyl aminopeptidase
1902	LPAR1	lysophosphatidic acid receptor 1
4023	LPL	lipoprotein lipase
116844	LRG1	leucine-rich alpha-2-glycoprotein 1
55791	LRIF1	ligand dependent nuclear receptor interacting factor 1
4035	LRP1	low density lipoprotein receptor-related protein 1
4043	LRPAP1	low density lipoprotein receptor-related protein associated protein 1
26065	LSM14A	LSM14A mRNA processing body assembly factor
4049	LTA	lymphotoxin alpha
4055	LTBR	lymphotoxin beta receptor (TNFR superfamily, member 3)
4067	LYN	LYN proto-oncogene, Src family tyrosine kinase

8216	LZTR1	leucine-zipper-like transcription regulator 1
23499	MACF1	microtubule-actin crosslinking factor 1
4099	MAG	myelin associated glycoprotein
9223	MAGI1	membrane associated guanylate kinase, WW and PDZ domain containing 1
260425	MAGI3	membrane associated guanylate kinase, WW and PDZ domain containing 3
84549	MAK16	MAK16 homolog
4130	MAP1A	microtubule-associated protein 1A
5605	MAP2K2	mitogen-activated protein kinase kinase 2
4214	MAP3K1	mitogen-activated protein kinase kinase kinase 1, E3 ubiquitin protein ligase
4216	MAP3K4	mitogen-activated protein kinase kinase kinase 4
6885	MAP3K7	mitogen-activated protein kinase kinase kinase 7
1432	MAPK14	mitogen-activated protein kinase 14
5597	MAPK6	mitogen-activated protein kinase 6
4145	MATK	megakaryocyte-associated tyrosine kinase
4153	MBL2	mannose-binding lectin (protein C) 2, soluble
4155	MBP	myelin basic protein
4163	MCC	mutated in colorectal cancers
4192	MDK	midkine (neurite growth-promoting factor 2)
5469	MED1	mediator complex subunit 1
23389	MED13L	mediator complex subunit 13-like
4223	MEOX2	mesenchyme homeobox 2
4224	MEP1A	meprin A, alpha (PABA peptide hydrolase)
4233	MET	MET proto-oncogene, receptor tyrosine kinase
256471	MFSD8	major facilitator superfamily domain containing 8
375056	MIA3	melanoma inhibitory activity family, member 3
100507436	MICA	MHC class I polypeptide-related sequence A
4277	MICB	MHC class I polypeptide-related sequence B
4291	MLF1	myeloid leukemia factor 1
4301	MLLT4	myeloid/lymphoid or mixed-lineage leukemia; translocated to, 4
4311	MME	membrane metallo-endopeptidase

4326	MMP17	matrix metallopeptidase 17 (membrane-inserted)
22880	MORC2	MORC family CW-type zinc finger 2
8777	MPDZ	multiple PDZ domain protein
10200	MPHOSPH6	M-phase phosphoprotein 6
51678	MPP6	membrane protein, palmitoylated 6 (MAGUK p55 subfamily member 6)
9902	MRC2	mannose receptor, C type 2
79922	MRM1	mitochondrial rRNA methyltransferase 1
79590	MRPL24	mitochondrial ribosomal protein L24
10573	MRPL28	mitochondrial ribosomal protein L28
128308	MRPL55	mitochondrial ribosomal protein L55
57380	MRS2	MRS2 magnesium transporter
345778	MTX3	metaxin 3
80198	MUS81	MUS81 structure-specific endonuclease subunit
23077	MYCBP2	MYC binding protein 2, E3 ubiquitin protein ligase
4615	MYD88	myeloid differentiation primary response 88
4619	MYH1	myosin, heavy chain 1, skeletal muscle, adult
8735	MYH13	myosin, heavy chain 13, skeletal muscle
4624	MYH6	myosin, heavy chain 6, cardiac muscle, alpha
80179	MYO19	myosin XIX
4641	MYO1C	myosin IC
64005	MYO1G	myosin IG
53904	MYO3A	myosin IIIA
4653	MYOC	myocilin, trabecular meshwork inducible glucocorticoid response
4654	MYOD1	myogenic differentiation 1
254827	NAALADL2	N-acetylated alpha-linked acidic dipeptidase-like 2
4677	NARS	asparaginyl-tRNA synthetase
9027	NAT8	N-acetyltransferase 8 (GCN5-related, putative)
29781	NCAPH2	non-SMC condensin II complex, subunit H2
4690	NCK1	NCK adaptor protein 1
8440	NCK2	NCK adaptor protein 2

56926	NCLN	nicalin
8648	NCOA1	nuclear receptor coactivator 1
10499	NCOA2	nuclear receptor coactivator 2
374383	NCR3LG1	natural killer cell cytotoxicity receptor 3 ligand 1
81565	NDEL1	nudE neurodevelopment protein 1-like 1
4716	NDUFB10	NADH dehydrogenase (ubiquinone) 1 beta subcomplex, 10, 22kDa
4711	NDUFB5	NADH dehydrogenase (ubiquinone) 1 beta subcomplex, 5, 16kDa
4726	NDUFS6	NADH dehydrogenase (ubiquinone) Fe-S protein 6, 13kDa (NADH-coenzyme Q reductase)
23327	NEDD4L	neural precursor cell expressed, developmentally down-regulated 4-like, E3 ubiquitin protein ligase
4747	NEFL	neurofilament, light polypeptide
10783	NEK6	NIMA-related kinase 6
140609	NEK7	NIMA-related kinase 7
284086	NEK8	NIMA-related kinase 8
4756	NEO1	neogenin 1
4790	NFKB1	nuclear factor of kappa light polypeptide gene enhancer in B-cells 1
4792	NFKBIA	nuclear factor of kappa light polypeptide gene enhancer in B-cells inhibitor, alpha
4803	NGF	nerve growth factor (beta polypeptide)
4804	NGFR	nerve growth factor receptor
4810	NHS	Nance-Horan syndrome (congenital cataracts and dental anomalies)
11188	NISCH	nischarin
28512	NKIRAS1	NFKB inhibitor interacting Ras-like 1
22871	NLGN1	neuroligin 1
57555	NLGN2	neuroligin 2
54413	NLGN3	neuroligin 3
4830	NME1	NME/NM23 nucleoside diphosphate kinase 1
654364	NME1-NME2	NME1-NME2 readthrough
4831	NME2	NME/NM23 nucleoside diphosphate kinase 2
4832	NME3	NME/NM23 nucleoside diphosphate kinase 3
65083	NOL6	nucleolar protein 6 (RNA-associated)
4839	NOP2	NOP2 nucleolar protein

4851	NOTCH1	notch 1
4869	NPM1	nucleophosmin (nucleolar phosphoprotein B23, numatrin)
190	NR0B1	nuclear receptor subfamily 0, group B, member 1
8829	NRP1	neuropilin 1
9379	NRXN2	neurexin 2
64324	NSD1	nuclear receptor binding SET domain protein 1
286053	NSMCE2	non-SMC element 2, MMS21 homolog (S. cerevisiae)
51559	NT5DC3	5'-nucleotidase domain containing 3
9423	NTN1	netrin 1
4914	NTRK1	neurotrophic tyrosine kinase, receptor, type 1
4915	NTRK2	neurotrophic tyrosine kinase, receptor, type 2
4916	NTRK3	neurotrophic tyrosine kinase, receptor, type 3
4922	NTS	neurotensin
81788	NUAK2	NUAK family, SNF1-like kinase, 2
23386	NUDCD3	NudC domain containing 3
79023	NUP37	nucleoporin 37kDa
8638	OASL	2'-5'-oligoadenylate synthetase-like
100506658	OCLN	occludin
4974	OMG	oligodendrocyte myelin glycoprotein
10133	OPTN	optineurin
10956	OS9	osteosarcoma amplified 9, endoplasmic reticulum lectin
130497	OSR1	odd-skipped related transciption factor 1
55611	OTUB1	OTU deubiquitinase, ubiquitin aldehyde binding 1
90268	OTULIN	OTU deubiquitinase with linear linkage specificity
9943	OXSR1	oxidative stress responsive 1
5021	OXTR	oxytocin receptor
5027	P2RX7	purinergic receptor P2X, ligand gated ion channel, 7
64805	P2RY12	purinergic receptor P2Y, G-protein coupled, 12
55690	PACS1	phosphofurin acidic cluster sorting protein 1
29763	PACSIN3	protein kinase C and casein kinase substrate in neurons 3

5058	PAK1	p21 protein (Cdc42/Rac)-activated kinase 1
79728	PALB2	partner and localizer of BRCA2
54873	PALMD	palmdelphin
24145	PANX1	pannexin 1
64282	PAPD5	PAP associated domain containing 5
50855	PARD6A	par-6 family cell polarity regulator alpha
5071	PARK2	parkin RBR E3 ubiquitin protein ligase
83666	PARP9	poly (ADP-ribose) polymerase family, member 9
23178	PASK	PAS domain containing serine/threonine kinase
5076	PAX2	paired box 2
5099	PCDH7	protocadherin 7
201626	PDE12	phosphodiesterase 12
5154	PDGFA	platelet-derived growth factor alpha polypeptide
5155	PDGFB	platelet-derived growth factor beta polypeptide
56034	PDGFC	platelet derived growth factor C
5156	PDGFRA	platelet-derived growth factor receptor, alpha polypeptide
5159	PDGFRB	platelet-derived growth factor receptor, beta polypeptide
149420	PDIK1L	PDLIM1 interacting kinase 1 like
79834	PEAK1	pseudopodium-enriched atypical kinase 1
5175	PECAM1	platelet/endothelial cell adhesion molecule 1
23089	PEG10	paternally expressed 10
57162	PELI1	pellino E3 ubiquitin protein ligase 1
57161	PELI2	pellino E3 ubiquitin protein ligase family member 2
246330	PELI3	pellino E3 ubiquitin protein ligase family member 3
1912	PHC2	polyhomeotic homolog 2 (Drosophila)
90102	PHLDB2	pleckstrin homology-like domain, family B, member 2
5297	PI4KA	phosphatidylinositol 4-kinase, catalytic, alpha
8554	PIAS1	protein inhibitor of activated STAT, 1
9063	PIAS2	protein inhibitor of activated STAT, 2
5295	PIK3R1	phosphoinositide-3-kinase, regulatory subunit 1 (alpha)

5296	PIK3R2	phosphoinositide-3-kinase, regulatory subunit 2 (beta)
8503	PIK3R3	phosphoinositide-3-kinase, regulatory subunit 3 (gamma)
5331	PLCB3	phospholipase C, beta 3 (phosphatidylinositol-specific)
5335	PLCG1	phospholipase C, gamma 1
5339	PLEC	plectin
5341	PLEK	pleckstrin
58473	PLEKHB1	pleckstrin homology domain containing, family B (evectins) member 1
55111	PLEKHJ1	pleckstrin homology domain containing, family J member 1
10733	PLK4	polo-like kinase 4
5362	PLXNA2	plexin A2
91584	PLXNA4	plexin A4
5371	PML	promyelocytic leukemia
10957	PNRC1	proline-rich nuclear receptor coactivator 1
55629	PNRC2	proline-rich nuclear receptor coactivator 2
134359	POC5	POC5 centriolar protein
23649	POLA2	polymerase (DNA directed), alpha 2, accessory subunit
84197	POMK	protein-O-mannose kinase
5445	PON2	paraoxonase 2
10940	POP1	POP1 homolog, ribonuclease P/MRP subunit
5451	POU2F1	POU class 2 homeobox 1
23759	PPIL2	peptidylprolyl isomerase (cyclophilin)-like 2
8493	PPM1D	protein phosphatase, Mg2+/Mn2+ dependent, 1D
5499	PPP1CA	protein phosphatase 1, catalytic subunit, alpha isozyme
26051	PPP1R16B	protein phosphatase 1, regulatory subunit 16B
5515	PPP2CA	protein phosphatase 2, catalytic subunit, alpha isozyme
5520	PPP2R2A	protein phosphatase 2, regulatory subunit B, alpha
55012	PPP2R3C	protein phosphatase 2, regulatory subunit B'', gamma
5533	PPP3CC	protein phosphatase 3, catalytic subunit, gamma isozyme
5536	PPP5C	protein phosphatase 5, catalytic subunit
5537	PPP6C	protein phosphatase 6, catalytic subunit

5546	PRCC	papillary renal cell carcinoma (translocation-associated)
10113	PREB	prolactin regulatory element binding
80243	PREX2	phosphatidylinositol-3,4,5-trisphosphate-dependent Rac exchange factor 2
144165	PRICKLE1	prickle homolog 1
5567	PRKACB	protein kinase, cAMP-dependent, catalytic, beta
5578	PRKCA	protein kinase C, alpha
5580	PRKCD	protein kinase C, delta
79706	PRKRIP1	PRKR interacting protein 1 (IL11 inducible)
5629	PROX1	prospero homeobox 1
9128	PRPF4	pre-mRNA processing factor 4
5657	PRTN3	proteinase 3
5663	PSEN1	presenilin 1
5690	PSMB2	proteasome (prosome, macropain) subunit, beta type, 2
5705	PSMC5	proteasome (prosome, macropain) 26S subunit, ATPase, 5
5708	PSMD2	proteasome (prosome, macropain) 26S subunit, non-ATPase, 2
5725	PTBP1	polypyrimidine tract binding protein 1
5728	PTEN	phosphatase and tensin homolog
5747	PTK2	protein tyrosine kinase 2
2185	PTK2B	protein tyrosine kinase 2 beta
5764	PTN	pleiotrophin
5770	PTPN1	protein tyrosine phosphatase, non-receptor type 1
5781	PTPN11	protein tyrosine phosphatase, non-receptor type 11
5777	PTPN6	protein tyrosine phosphatase, non-receptor type 6
5788	PTPRC	protein tyrosine phosphatase, receptor type, C
5802	PTPRS	protein tyrosine phosphatase, receptor type, S
5803	PTPRZ1	protein tyrosine phosphatase, receptor-type, Z polypeptide 1
54899	PXK	PX domain containing serine/threonine kinase
5829	PXN	paxillin
80223	RAB11FIP1	RAB11 family interacting protein 1 (class I)
26056	RAB11FIP5	RAB11 family interacting protein 5 (class I)

5872	RAB13	RAB13, member RAS oncogene family
22931	RAB18	RAB18, member RAS oncogene family
57111	RAB25	RAB25, member RAS oncogene family
83452	RAB33B	RAB33B, member RAS oncogene family
83871	RAB34	RAB34, member RAS oncogene family
23682	RAB38	RAB38, member RAS oncogene family
5865	RAB3B	RAB3B, member RAS oncogene family
5868	RAB5A	RAB5A, member RAS oncogene family
5869	RAB5B	RAB5B, member RAS oncogene family
5878	RAB5C	RAB5C, member RAS oncogene family
51560	RAB6B	RAB6B, member RAS oncogene family
7879	RAB7A	RAB7A, member RAS oncogene family
4218	RAB8A	RAB8A, member RAS oncogene family
5877	RABIF	RAB interacting factor
55698	RADIL	Ras association and DIL domains
8480	RAE1	ribonucleic acid export 1
135250	RAET1E	retinoic acid early transcript 1E
353091	RAET1G	retinoic acid early transcript 1G
5894	RAF1	Raf-1 proto-oncogene, serine/threonine kinase
10928	RALBP1	ralA binding protein 1
10267	RAMP1	receptor (G protein-coupled) activity modifying protein 1
10268	RAMP3	receptor (G protein-coupled) activity modifying protein 3
5901	RAN	RAN, member RAS oncogene family
10048	RANBP9	RAN binding protein 9
5911	RAP2A	RAP2A, member of RAS oncogene family
23551	RASD2	RASD family, member 2
51285	RASL12	RAS-like, family 12
9821	RB1CC1	RB1-inducible coiled-coil 1
10616	RBCK1	RanBP-type and C3HC4-type zinc finger containing 1
55131	RBM28	RNA binding motif protein 28

11030	RBPMS	RNA binding protein with multiple splicing
5979	RET	ret proto-oncogene
91869	RFT1	RFT1 homolog
6002	RGS12	regulator of G-protein signaling 12
10287	RGS19	regulator of G-protein signaling 19
6009	RHEB	Ras homolog enriched in brain
121268	RHEBL1	Ras homolog enriched in brain like 1
387	RHOA	ras homolog family member A
29984	RHOD	ras homolog family member D
391	RHOG	ras homolog family member G
399	RHOH	ras homolog family member H
57381	RHOJ	ras homolog family member J
85415	RHPN2	rhophilin, Rho GTPase binding protein 2
8737	RIPK1	receptor (TNFRSF)-interacting serine-threonine kinase 1
55005	RMND1	required for meiotic nuclear division 1 homolog (S. cerevisiae)
55819	RNF130	ring finger protein 130
81847	RNF146	ring finger protein 146
81790	RNF170	ring finger protein 170
6045	RNF2	ring finger protein 2
54476	RNF216	ring finger protein 216
55072	RNF31	ring finger protein 31
9810	RNF40	ring finger protein 40, E3 ubiquitin protein ligase
6048	RNF5	ring finger protein 5, E3 ubiquitin protein ligase
9025	RNF8	ring finger protein 8, E3 ubiquitin protein ligase
10921	RNPS1	RNA binding protein S1, serine-rich domain
6095	RORA	RAR-related orphan receptor A
6098	ROS1	ROS proto-oncogene 1 , receptor tyrosine kinase
6117	RPA1	replication protein A1, 70kDa
6118	RPA2	replication protein A2, 32kDa
6119	RPA3	replication protein A3, 14kDa

79657	RPAP3	RNA polymerase II associated protein 3
23322	RPGRIP1L	RPGRIP1-like
6132	RPL8	ribosomal protein L8
6222	RPS18	ribosomal protein S18
6233	RPS27A	ribosomal protein S27a
6197	RPS6KA3	ribosomal protein S6 kinase, 90kDa, polypeptide 3
22800	RRAS2	related RAS viral (r-ras) oncogene homolog 2
10692	RRH	retinal pigment epithelium-derived rhodopsin homolog
23076	RRP1B	ribosomal RNA processing 1B
284654	RSPO1	R-spondin 1
6242	RTKN	rhotekin
57142	RTN4	reticulon 4
84816	RTN4IP1	reticulon 4 interacting protein 1
65078	RTN4R	reticulon 4 receptor
8607	RUVBL1	RuvB-like AAA ATPase 1
6256	RXRA	retinoid X receptor, alpha
6259	RYK	receptor-like tyrosine kinase
9294	S1PR2	sphingosine-1-phosphate receptor 2
1903	S1PR3	sphingosine-1-phosphate receptor 3
113174	SAAL1	serum amyloid A-like 1
22908	SACM1L	SAC1 suppressor of actin mutations 1-like (yeast)
142891	SAMD8	sterile alpha motif domain containing 8
23328	SASH1	SAM and SH3 domain containing 1
54440	SASH3	SAM and SH3 domain containing 3
112483	SAT2	spermidine/spermine N1-acetyltransferase family member 2
81846	SBF2	SET binding factor 2
23256	SCFD1	sec1 family domain containing 1
7857	SCG2	secretogranin II
6386	SDCBP	syndecan binding protein (syntenin)
9919	SEC16A	SEC16 homolog A (S. cerevisiae)

29927	SEC61A1	Sec61 alpha 1 subunit (S. cerevisiae)
6400	SEL1L	sel-1 suppressor of lin-12-like (C. elegans)
6403	SELP	selectin P (granule membrane protein 140kDa, antigen CD62)
6404	SELPLG	selectin P ligand
54910	SEMA4C	sema domain, immunoglobulin domain (Ig), transmembrane domain (TM) and short cytoplasmic domain, (semaphorin) 4C
26168	SENP3	SUMO1/sentrin/SMT3 specific peptidase 3
710	SERPING1	serpin peptidase inhibitor, clade G (C1 inhibitor), member 1
94097	SFXN5	sideroflexin 5
6449	SGTA	small glutamine-rich tetratricopeptide repeat (TPR)-containing, alpha
54557	SGTB	small glutamine-rich tetratricopeptide repeat (TPR)-containing, beta
25970	SH2B1	SH2B adaptor protein 1
23677	SH3BP4	SH3-domain binding protein 4
30011	SH3KBP1	SH3-domain kinase binding protein 1
285590	SH3PXD2B	SH3 and PX domains 2B
81858	SHARPIN	SHANK-associated RH domain interactor
6461	SHB	Src homology 2 domain containing adaptor protein B
6464	SHC1	SHC (Src homology 2 domain containing) transforming protein 1
53358	SHC3	SHC (Src homology 2 domain containing) transforming protein 3
90525	SHF	Src homology 2 domain containing F
23408	SIRT5	sirtuin 5
8935	SKAP2	src kinase associated phosphoprotein 2
6497	SKI	SKI proto-oncogene
6498	SKIL	SKI-like proto-oncogene
114789	SLC25A25	solute carrier family 25 (mitochondrial carrier; phosphate carrier), member 25
293	SLC25A6	solute carrier family 25 (mitochondrial carrier; adenine nucleotide translocator), member 6
148867	SLC30A7	solute carrier family 30 (zinc transporter), member 7
27173	SLC39A1	solute carrier family 39 (zinc transporter), member 1
9368	SLC9A3R1	solute carrier family 9, subfamily A (NHE3, cation proton antiporter 3), member 3 regulator 1
10569	SLU7	SLU7 splicing factor homolog (S. cerevisiae)
4086	SMAD1	SMAD family member 1

4087	SMAD2	SMAD family member 2
4088	SMAD3	SMAD family member 3
4089	SMAD4	SMAD family member 4
4091	SMAD6	SMAD family member 6
4092	SMAD7	SMAD family member 7
6604	SMARCD3	SWI/SNF related, matrix associated, actin dependent regulator of chromatin, subfamily d, member 3
9887	SMG7	SMG7 nonsense mediated mRNA decay factor
55512	SMPD3	sphingomyelin phosphodiesterase 3, neutral membrane (neutral sphingomyelinase II)
57154	SMURF1	SMAD specific E3 ubiquitin protein ligase 1
64750	SMURF2	SMAD specific E3 ubiquitin protein ligase 2
6640	SNTA1	syntrophin, alpha 1
54212	SNTG1	syntrophin, gamma 1
6642	SNX1	sorting nexin 1
29916	SNX11	sorting nexin 11
9784	SNX17	sorting nexin 17
6643	SNX2	sorting nexin 2
81609	SNX27	sorting nexin family member 27
8723	SNX4	sorting nexin 4
58533	SNX6	sorting nexin 6
8651	SOCS1	suppressor of cytokine signaling 1
9021	SOCS3	suppressor of cytokine signaling 3
9306	SOCS6	suppressor of cytokine signaling 6
114815	SORCS1	sortilin-related VPS10 domain containing receptor 1
6653	SORL1	sortilin-related receptor, L(DLR class) A repeats containing
6272	SORT1	sortilin 1
80320	SP6	Sp6 transcription factor
8404	SPARCL1	SPARC-like 1 (hevin)
10653	SPINT2	serine peptidase inhibitor, Kunitz type, 2
6695	SPOCK1	sparc/osteonectin, cwcv and kazal-like domains proteoglycan (testican) 1
6696	SPP1	secreted phosphoprotein 1

84888	SPPL2A	signal peptide peptidase like 2A
6711	SPTBN1	spectrin, beta, non-erythrocytic 1
6712	SPTBN2	spectrin, beta, non-erythrocytic 2
8878	SQSTM1	sequestosome 1
6714	SRC	SRC proto-oncogene, non-receptor tyrosine kinase
6721	SREBF2	sterol regulatory element binding transcription factor 2
57522	SRGAP1	SLIT-ROBO Rho GTPase activating protein 1
6732	SRPK1	SRSF protein kinase 1
6733	SRPK2	SRSF protein kinase 2
6427	SRSF2	serine/arginine-rich splicing factor 2
54961	SSH3	slingshot protein phosphatase 3
6745	SSR1	signal sequence receptor, alpha
6767	ST13	suppression of tumorigenicity 13 (colon carcinoma) (Hsp70 interacting protein)
8027	STAM	signal transducing adaptor molecule (SH3 domain and ITAM motif) 1
10617	STAMBP	STAM binding protein
6772	STAT1	signal transducer and activator of transcription 1, 91kDa
6774	STAT3	signal transducer and activator of transcription 3 (acute-phase response factor)
6776	STAT5A	signal transducer and activator of transcription 5A
6777	STAT5B	signal transducer and activator of transcription 5B
140901	STK35	serine/threonine kinase 35
11171	STRAP	serine/threonine kinase receptor associated protein
10273	STUB1	STIP1 homology and U-box containing protein 1, E3 ubiquitin protein ligase
9482	STX8	syntaxin 8
51657	STYXL1	serine/threonine/tyrosine interacting-like 1
6840	SVIL	supervillin
258010	SVIP	small VCP/p97-interacting protein
6850	SYK	spleen tyrosine kinase
10492	SYNCRIP	synaptotagmin binding, cytoplasmic RNA interacting protein
163183	SYNE4	spectrin repeat containing, nuclear envelope family member 4
10454	TAB1	TGF-beta activated kinase 1/MAP3K7 binding protein 1

23118	TAB2	TGF-beta activated kinase 1/MAP3K7 binding protein 2
132001	TAMM41	TAM41, mitochondrial translocator assembly and maintenance protein, homolog (S. cerevisiae)
57533	TBC1D14	TBC1 domain family, member 14
79735	TBC1D17	TBC1 domain family, member 17
29110	TBK1	TANK-binding kinase 1
9519	TBPL1	TBP-like 1
10312	TCIRG1	T-cell, immune regulator 1, ATPase, H+ transporting, lysosomal V0 subunit A3
6949	TCOF1	Treacher Collins-Franceschetti syndrome 1
26123	TCTN3	tectonic family member 3
6997	TDGF1	teratocarcinoma-derived growth factor 1
7006	TEC	tec protein tyrosine kinase
7018	TF	transferrin
7022	TFAP2C	transcription factor AP-2 gamma (activating enhancer binding protein 2 gamma)
7037	TFRC	transferrin receptor
7040	TGFB1	transforming growth factor, beta 1
7042	TGFB2	transforming growth factor, beta 2
7043	TGFB3	transforming growth factor, beta 3
7046	TGFBR1	transforming growth factor, beta receptor 1
7048	TGFBR2	transforming growth factor, beta receptor II (70/80kDa)
9392	TGFBRAP1	transforming growth factor, beta receptor associated protein 1
7057	THBS1	thrombospondin 1
8563	THOC5	THO complex 5
353376	TICAM2	toll-like receptor adaptor molecule 2
114609	TIRAP	toll-interleukin 1 receptor (TIR) domain containing adaptor protein
7094	TLN1	talin 1
7099	TLR4	toll-like receptor 4
51284	TLR7	toll-like receptor 7
80213	TM2D3	TM2 domain containing 3
10548	TM9SF1	transmembrane 9 superfamily member 1
11018	TMED1	transmembrane emp24 protein transport domain containing 1

100302736	TMED7-TICAM2	TMED7-TICAM2 readthrough
153396	TMEM161B	transmembrane protein 161B
84187	TMEM164	transmembrane protein 164
200728	TMEM17	transmembrane protein 17
51259	TMEM216	transmembrane protein 216
79583	TMEM231	transmembrane protein 231
91304	TMEM259	transmembrane protein 259
55754	TMEM30A	transmembrane protein 30A
55116	TMEM39B	transmembrane protein 39B
252839	TMEM9	transmembrane protein 9
643853	TMPPE	transmembrane protein with metallophosphoesterase domain
3371	TNC	tenascin C
7124	TNF	tumor necrosis factor
7128	TNFAIP3	tumor necrosis factor, alpha-induced protein 3
23495	TNFRSF13B	tumor necrosis factor receptor superfamily, member 13B
115650	TNFRSF13C	tumor necrosis factor receptor superfamily, member 13C
7132	TNFRSF1A	tumor necrosis factor receptor superfamily, member 1A
7133	TNFRSF1B	tumor necrosis factor receptor superfamily, member 1B
10673	TNFSF13B	tumor necrosis factor (ligand) superfamily, member 13b
8744	TNFSF9	tumor necrosis factor (ligand) superfamily, member 9
10318	TNIP1	TNFAIP3 interacting protein 1
8711	TNK1	tyrosine kinase, non-receptor, 1
10188	TNK2	tyrosine kinase, non-receptor, 2
7138	TNNT1	troponin T type 1 (skeletal, slow)
23534	TNPO3	transportin 3
7143	TNR	tenascin R
27327	TNRC6A	trinucleotide repeat containing 6A
23371	TNS2	tensin 2
54472	TOLLIP	toll interacting protein

7157	TP53	tumor protein p53
7168	TPM1	tropomyosin 1 (alpha)
8717	TRADD	TNFRSF1A-associated via death domain
7185	TRAF1	TNF receptor-associated factor 1
7186	TRAF2	TNF receptor-associated factor 2
7187	TRAF3	TNF receptor-associated factor 3
26146	TRAF3IP1	TNF receptor-associated factor 3 interacting protein 1
10758	TRAF3IP2	TRAF3 interacting protein 2
9618	TRAF4	TNF receptor-associated factor 4
7189	TRAF6	TNF receptor-associated factor 6, E3 ubiquitin protein ligase
10131	TRAP1	TNF receptor-associated protein 1
11078	TRIOBP	TRIO and F-actin binding protein
7205	TRIP6	thyroid hormone receptor interactor 6
7220	TRPC1	transient receptor potential cation channel, subfamily C, member 1
8848	TSC22D1	TSC22 domain family, member 1
80746	TSEN2	TSEN2 tRNA splicing endonuclease subunit
7257	TSNAX	translin-associated factor X
23555	TSPAN15	tetraspanin 15
26262	TSPAN17	tetraspanin 17
10099	TSPAN3	tetraspanin 3
83942	TSSK1B	testis-specific serine kinase 1B
283629	TSSK4	testis-specific serine kinase 4
7265	TTC1	tetratricopeptide repeat domain 1
55761	TTC17	tetratricopeptide repeat domain 17
55622	TTC27	tetratricopeptide repeat domain 27
22996	TTC39A	tetratricopeptide repeat domain 39A
79183	TTPAL	tocopherol (alpha) transfer protein-like
80727	TTYH3	tweety family member 3
84790	TUBA1C	tubulin, alpha 1c
203068	TUBB	tubulin, beta class I

51061	TXNDC11	thioredoxin domain containing 11
84817	TXNDC17	thioredoxin domain containing 17
7297	TYK2	tyrosine kinase 2
7301	TYRO3	TYRO3 protein tyrosine kinase
7305	TYROBP	TYRO protein tyrosine kinase binding protein
55075	UACA	uveal autoantigen with coiled-coil domains and ankyrin repeats
6675	UAP1	UDP-N-acetylglucosamine pyrophosphorylase 1
7311	UBA52	ubiquitin A-52 residue ribosomal protein fusion product 1
51271	UBAP1	ubiquitin associated protein 1
7314	UBB	ubiquitin B
7316	UBC	ubiquitin C
10537	UBD	ubiquitin D
10477	UBE2E3	ubiquitin-conjugating enzyme E2E 3
7334	UBE2N	ubiquitin-conjugating enzyme E2N
65264	UBE2Z	ubiquitin-conjugating enzyme E2Z
29979	UBQLN1	ubiquilin 1
56893	UBQLN4	ubiquilin 4
51035	UBXN1	UBX domain protein 1
7349	UCN	urocortin
90226	UCN2	urocortin 2
114131	UCN3	urocortin 3
23376	UFL1	UFM1-specific ligase 1
55325	UFSP2	UFM1-specific peptidase 2
55757	UGGT2	UDP-glucose glycoprotein glucosyltransferase 2
80328	ULBP2	UL16 binding protein 2
79465	ULBP3	UL16 binding protein 3
8633	UNC5C	unc-5 netrin receptor C
5976	UPF1	UPF1 regulator of nonsense transcripts homolog (yeast)
9875	URB1	URB1 ribosome biogenesis 1 homolog (S. cerevisiae)
9816	URB2	URB2 ribosome biogenesis 2 homolog (S. cerevisiae)

7398	USP1	ubiquitin specific peptidase 1
8237	USP11	ubiquitin specific peptidase 11
9958	USP15	ubiquitin specific peptidase 15
84669	USP32	ubiquitin specific peptidase 32
7375	USP4	ubiquitin specific peptidase 4 (proto-oncogene)
85015	USP45	ubiquitin specific peptidase 45
84135	UTP15	UTP15, U3 small nucleolar ribonucleoprotein, homolog (S. cerevisiae)
10451	VAV3	vav 3 guanine nucleotide exchange factor
7412	VCAM1	vascular cell adhesion molecule 1
1462	VCAN	versican
7415	VCP	valosin containing protein
79674	VEPH1	ventricular zone expressed PH domain-containing 1
7431	VIM	vimentin
7432	VIP	vasoactive intestinal peptide
7433	VIPR1	vasoactive intestinal peptide receptor 1
7434	VIPR2	vasoactive intestinal peptide receptor 2
154807	VKORC1L1	vitamin K epoxide reductase complex, subunit 1-like 1
7436	VLDLR	very low density lipoprotein receptor
9730	VPRBP	Vpr (HIV-1) binding protein
64601	VPS16	vacuolar protein sorting 16 homolog (S. cerevisiae)
738	VPS51	vacuolar protein sorting 51 homolog (S. cerevisiae)
7454	WAS	Wiskott-Aldrich syndrome
64743	WDR13	WD repeat domain 13
55339	WDR33	WD repeat domain 33
80349	WDR61	WD repeat domain 61
54663	WDR74	WD repeat domain 74
8838	WISP3	WNT1 inducible signaling pathway protein 3
7473	WNT3	wingless-type MMTV integration site family, member 3
11059	WWP1	WW domain containing E3 ubiquitin protein ligase 1
11060	WWP2	WW domain containing E3 ubiquitin protein ligase 2

331	XIAP	X-linked inhibitor of apoptosis, E3 ubiquitin protein ligase
64328	XPO4	exportin 4
7516	XRCC2	X-ray repair complementing defective repair in Chinese hamster cells 2
7517	XRCC3	X-ray repair complementing defective repair in Chinese hamster cells 3
8565	YARS	tyrosyl-tRNA synthetase
4904	YBX1	Y box binding protein 1
7525	YES1	YES proto-oncogene 1, Src family tyrosine kinase
25844	YIPF3	Yip1 domain family, member 3
7529	YWHAB	tyrosine 3-monooxygenase/tryptophan 5-monooxygenase activation protein, beta
10971	YWHAQ	tyrosine 3-monooxygenase/tryptophan 5-monooxygenase activation protein, theta
7534	YWHAZ	tyrosine 3-monooxygenase/tryptophan 5-monooxygenase activation protein, zeta
7535	ZAP70	zeta-chain (TCR) associated protein kinase 70kDa
7597	ZBTB25	zinc finger and BTB domain containing 25
56829	ZC3HAV1	zinc finger CCCH-type, antiviral 1
286128	ZFP41	ZFP41 zinc finger protein
9372	ZFYVE9	zinc finger, FYVE domain containing 9
84146	ZNF644	zinc finger protein 644
79724	ZNF768	zinc finger protein 768
79364	ZXDC	ZXD family zinc finger C
